# PHEX Mimetic (SPR4-Peptide) Corrects and Improves HYP and Wild Type Mice Energy-Metabolism

**DOI:** 10.1371/journal.pone.0097326

**Published:** 2014-05-19

**Authors:** Lesya V. Zelenchuk, Anne-Marie Hedge, Peter S. N. Rowe

**Affiliations:** Internal Medicine, The Kidney Institute, Kansas University Medical Center (KUMC), Kansas City, Kansas, United States of America; Faculté de médecine de Nantes, France

## Abstract

**Context:**

PHEX or DMP1 mutations cause hypophosphatemic-rickets and altered energy metabolism. PHEX binds to DMP1-ASARM-motif to form a complex with α_5_β_3_ integrin that suppresses FGF23 expression. ASARM-peptides increase FGF23 by disrupting the PHEX-DMP1-Integrin complex. We used a 4.2 kDa peptide (SPR4) that binds to ASARM-peptide/motif to study the DMP1-PHEX interaction and to assess SPR4 for the treatment of energy metabolism defects in HYP and potentially other bone-mineral disorders.

**Design:**

Subcutaneously transplanted osmotic pumps were used to infuse SPR4-peptide or vehicle (VE) into wild-type mice (WT) and HYP-mice (PHEX mutation) for 4 weeks.

**Results:**

SPR4 partially corrected HYP mice hypophosphatemia and increased serum 1.25(OH)_2_D_3_. Serum FGF23 remained high and PTH was unaffected. WT-SPR4 mice developed hypophosphatemia and hypercalcemia with increased PTH, FGF23 and 1.25(OH)_2_D_3_. SPR4 increased GAPDH HYP-bone expression 60× and corrected HYP-mice hyperglycemia and hypoinsulinemia. HYP-VE serum uric-acid (UA) levels were reduced and SPR4 infusion suppressed UA levels in WT-mice but not HYP-mice. SPR4 altered leptin, adiponectin, and sympathetic-tone and increased the fat mass/weight ratio for HYP and WT mice. Expression of perlipin-2 a gene involved in obesity was reduced in HYP-VE and WT-SPR4 mice but increased in HYP-SPR4 mice. Also, increased expression of two genes that inhibit insulin-signaling, ENPP1 and ESP, occurred with HYP-VE mice. In contrast, SPR4 reduced expression of both ENPP1 and ESP in WT mice and suppressed ENPP1 in HYP mice. Increased expression of FAM20C and sclerostin occurred with HYP-VE mice. SPR4 suppressed expression of FAM20C and sclerostin in HYP and WT mice.

**Conclusions:**

ASARM peptides and motifs are physiological substrates for PHEX and modulate osteocyte PHEX-DMP1-α_5_β_3_-integrin interactions and thereby FGF23 expression. These interactions also provide a nexus that regulates bone and energy metabolism. SPR4 suppression of sclerostin and/or sequestration of ASARM-peptides improves energy metabolism and may have utility for treating familial rickets, osteoporosis, obesity and diabetes.

## Introduction

Studies carried out by the Centers for Disease Control (CDC) confirm that approximately 70% of US adults were obese or overweight from 2009 to 2010. Of these nearly 40% were classed as overtly obese. Osteoporosis, a component of the metabolic syndrome that is associated with dyslipidemia and obesity [1], is also a major health issue for more than 44 million Americans. The cost for osteoporosis and related fractures total $14 billion per year. The social and financial cost to society is incalculable and growing at an exponential rate. Several genome wide association studies have confirmed MEPE as a major gene locus for bone mineral density and osteoporosis [2]–[6]. Also, serum levels of MEPE in normal humans correlates with serum phosphorus, parathyroid hormone and bone mineral density (BMD) [7], [8] Recent research has begun to unravel the intricacies of the molecular and physiological pathways linking energy metabolism and bone-renal mineral metabolism. This novel approach has exploited transgenic mice models and the new paradigm has been heralded as integrative physiology [9]. Thus far, three key physiological pathways have emerged. First, bone formation and resorption are proposed to regulate blood glucose via a feedback loop controlled by insulin and a bone matrix protein osteocalcin. Second, this pathway is also impacted by an adipokine leptin that can traverse the blood brain barrier and regulate biosynthesis of a neurotransmitter serotonin. The serotonergic signaling in the brainstem is proposed to affect sympathetic tone in the arcuate nucleus (AN) and ventrolateral medial nucleus (VMN) of the hypothalamus. Thus, AN serotonergic signaling increases appetite and VMH serotonergic-signaling decreases “bone resorption” and increases “bone formation” via sympathetic activation of osteoblast β-adrenergic receptors. Third, additional complexity has arisen with the discovery that circulating gut derived serotonin whose biosynthesis is regulated by Lrp5 negatively regulates bone formation. Serotonin does not cross the blood-brain barrier so the two distinct central and peripheral pools of serotonin are proposed to have opposite effects on bone turnover. Other neuropeptides also play key roles. For example brain expressed Cocaine Amphetamine Related Transcript (CART) is an inhibitor of bone resorption. The evidence for these pathways is compelling and the experimental science used to formulate the hypotheses elegant [10]–[20]. However, recent equally compelling and scientifically rigorous studies contradict all three pathways and so the models remain controversial [21]–[34].

Two new broad areas of study that ostensibly have arisen separately may provide answers to the contradictions and differing observations. First, the unraveling of novel molecular mechanisms regulating phosphate bone-mineral metabolism and the discovery of key genes involved in several inherited bone mineral loss disorders. A group of these genes are responsible for several inherited diseases causing hypophosphatemic rickets [35]. These diseases are unified further by dis-regulated FGF23 expression and profound changes in energy metabolism. Second, the wealth of phylogenetic information from differing species, evolutionary paleoanthropological studies, reconstruction of ancient geological landscapes, climates and associated flora and fauna. For example, the discovery that two energy metabolism genes, uricase and L-gulono lactone oxidase that are expressed in all mammals were selectively knocked out in ancient primates in the Miocene and Eocene epochs provides a clue [36]. Specifically, uric acid, (degraded by uricase) and vitamin C (synthesized by L-gulono lactone oxidase) have been shown to be closely associated with osteoporosis, chronic kidney disease, cardiovascular-disease, metabolic syndrome, diabetes and energy-metabolism in man [37]–[47].

In these studies we have used mice with a defect in the PHEX gene (HYP-mice) [48]. These mice are murine homologs for X-linked hypophosphatemic rickets (HYP) and are also hyperglycemic, hypoinsulinemic with altered energy metabolism [49]–[53]. The physiological substrate for PHEX is free ASARM-peptide or the ASARM-motif [54]–[66]. The ASARM-motif is an integral peptide sequence motif that is present in a group of extracellular matrix proteins called SIBLINGs. These SIBLINGs include DMP1, MEPE, DSPP, Osteopontin, Statherin and BSP [67]. Mutations in DMP-1 are responsible for autosomal recessive hypophosphatemic rickets (ARHR-1) a disease that has an overlapping pathophysiology with HYP [68], [69]. In both ARHR-1 and HYP increased circulating ASARM-peptides contribute to the mineralization defect and hyperosteoidosis [7], [54], [55], [58]–[66], [70], [71]. Compelling evidence indicates osteocyte membrane-bound PHEX binds to DMP1-ASARM-motif to form a hetero-complex with α_5_β_3_ integrin that suppresses FGF23 expression [67]. In our previous studies we designed a small 4.2 kDa PHEX related peptide (SPR4) that specifically binds to ASARM-peptide and ASARM-motif to study PHEX, DMP1, integrin and ASARM-peptide interactions and activities [56], [66]. This peptide (SPR4) has potent biological activity *in vitro* and is both a competitive inhibitor of PHEX activity and also neutralizes ASARM-peptide activity (inhibitor of mineralization) [56], [66]. In this study we used osmotic pumps to infuse SPR4-peptide into HYP and WT male mice for 4 weeks. SPR4 partially corrected HYP mice hypophosphatemia and increased 1.25(OH)_2_D_3_. FGF23 however, remained abnormally high and although a trend of serum PTH suppression was measured this was not significant. WT-SPR4 mice developed hypophosphatemia and hypercalcemia with increased PTH, FGF23 and 1.25(OH)_2_D_3_. Also, SPR4 increased GAPDH bone expression 60× fold and corrected HYP-mice hyperglycemia and hypoinsulinemia. Vehicle treated HYP mice (HYP-VE) serum uric acid levels (UA) were reduced markedly relative to WT-VE mice. SPR4 infusion however, suppressed uric-acid levels in WT-SPR4 mice but not HYP-SPR4 mice. Also, SPR4 induced major changes in leptin, adiponectin, sympathetic tone and increased the fat mass/weight ratio for both HYP and WT mice. Expression of perlipin-2 (Plin-2) a gene involved in diet induced obesity and adipose inflammation was reduced in HYP-VE and WT-SPR4 mice but increased in HYP-SPR4 mice. Also, increased expression of two genes that inhibit insulin signaling, Ectonucleotide Pyrophosphatase Phosphodiesterase (ENPP1) and Osteotesticular Protein Tyrosine Phosphatase (OST-PTP or ESP) occurred with control vehicle HYP mice (HYP-VE) relative to control vehicle WT mice (WT-VE). In contrast, SPR4 infusion reduced expression of both ENPP1 and ESP in WT mice and suppressed ENPP1 in HYP mice. An increase in FAM20C kinase expression, a bone “ASARM-motif” specific kinase responsible for a form of autosomal recessive hypophosphatemic rickets (ARHR 2) [72]–[75] and sclerostin occurred with HYP mice relative to WT mice. SPR4 treatment of both HYP and WT mice markedly suppressed expression of FAM20C and sclerostin (protein and mRNA). These SPR4-peptide induced changes coincided with improved and corrected energy metabolism with WT and HYP mice respectively. This approach for the first time demonstrated the *in vivo* effects of the competitive inhibition of PHEX (WT-mice) and the sequestration of ASARM-peptide PHEX-substrate (HYP mice) in WT and HYP mice respectively.

We conclude that PHEX signaling orchestrates energy metabolism and interactions between the ASARM-motif of DMP1 and ASARM-peptides play a major role. These interactions in turn likely involve PHEX, DMP1 and α_5_β_3_ integrin cell surface regulation and signaling of FGF23. Chronic infusion of SPR4-peptide over 4 weeks produced marked improvements in mineral and energy metabolism in HYP mice. Specifically, SPR4 treatment induced a correction of the HYP mice hyperglycemia, hypoinsulinemia, hypoleptinemia, adiponectinemia and increased the percentage fat mass weight ratio with HYP and WT mice. This was accompanied by a marked suppression ENPP1 and FAM20C expression in SPR4 treated HYP and WT mice and ESP in WT mice. Our experiments also show SPR4-peptide infusion induces changes in Wild type mice that mimic aspects of the HYP mice phenotype (suppressed FGF23, PTH with hypophosphatemia). This pharmacologic bimodality likely reflects the dual nature and kinetics of SPR4-peptide activity. Specifically: (1) the competitive inhibition of PHEX-DMP1-integrin binding and commensurate altered FGF23 expression, and (2) the direct sequestration of the PHEX substrate, ASARM-peptide. The SPR4-peptide mediated suppression of sclerostin and improvement in energy metabolism may have therapeutic utility for osteoporosis, obesity and diabetes.

## Results

### Bone-renal biomarkers: vehicle treated HYP mice versus WT mice and increased sclerostin

**Figure 1:**
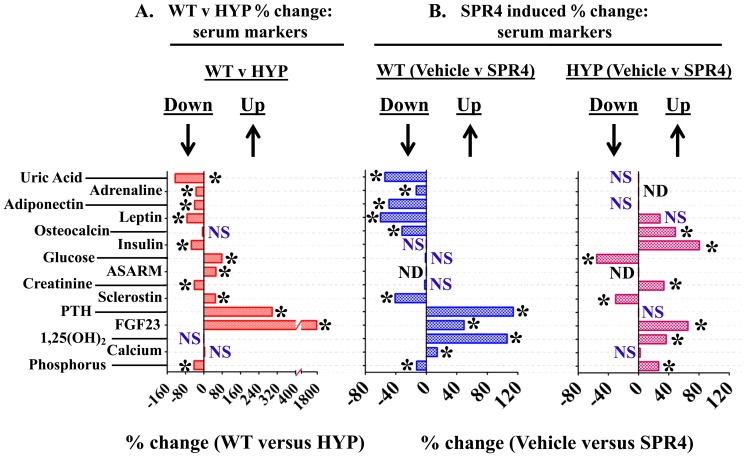
Percentage difference serum chemistry comparisons between wild type (WT) and HYP mice and mice infused with vehicle or SPR4-peptide. For absolute measurements in tabulated form see **Table 1**. Mice were sacrificed on day 28 and sera prepared from 16 hour fasted mice housed in metabolic cages. Values are means of percentage difference and are significant (* = P<0.05) unless indicated by NS (unpaired t test, confidence interval  = 95%; see Table 2 for absolute numbers). Column headings represent: **WT**  =  wild type mice; **HYP**  =  X-linked hypophosphatemic rickets mice; **SPR4**  =  infused SPR4-peptide; **Vehicle**  =  Saline infused; **NS**  =  not significant; **ND**  =  not done; * = P<0.05. Histogram bars to the left of zero on the axis indicate down regulation and to the right up regulation.

**Figure 2:**
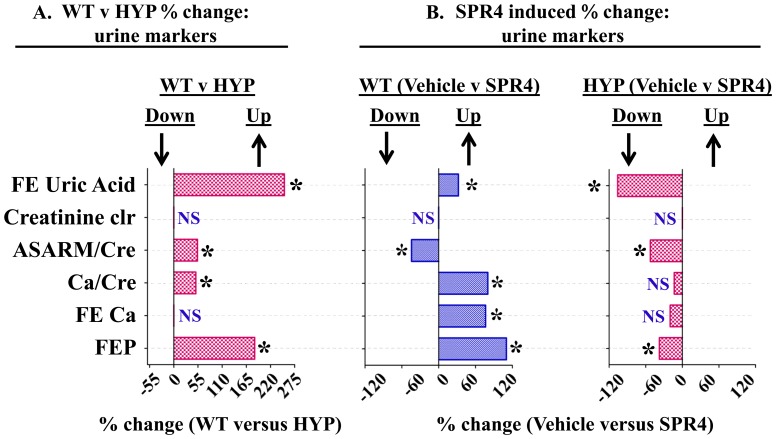
Percentage difference urine chemistry comparisons between wild type (WT) and HYP mice and mice infused with vehicle or SPR4-peptide. For absolute measurements in tabulated form see **Table 2**. Mice were sacrificed on day 28 and urine collected from 16 hour fasted mice housed in metabolic cages. Values are means of percentage difference and are significant (* = P<0.05) unless indicated by NS (unpaired t test confidence, interval  = 95%; see also Table 3 for absolute numbers). Column headings represent; **WT**  =  wild type mice, **HYP**  =  X-linked hypophosphatemic rickets mice, **SPR4**  =  infused SPR4-peptide and **Vehicle**  =  Saline infused. Histogram bars to the left of zero on the axis indicate down regulation and to the right up regulation. ***Index*** : **FE Uric Acid**  =  percentage change fractional Excretion of uric acid; **creatinine clearance**  =  percentage change creatinine clearance; **ASARM/Cre**  =  percentage change in ASARM/creatinine; **Ca/Cre**  =  percentage change in calcium/creatinine ratio; **Fe Ca**  =  percentage change in the fractional excretion of calcium; **FEP**  =  percentage change in the fractional excretion of phosphate; **NS**  =  not significant; * = P<0.05. Histogram bars to the left of zero on the axis indicate down regulation and to the right up regulation.

**Figure 3:**
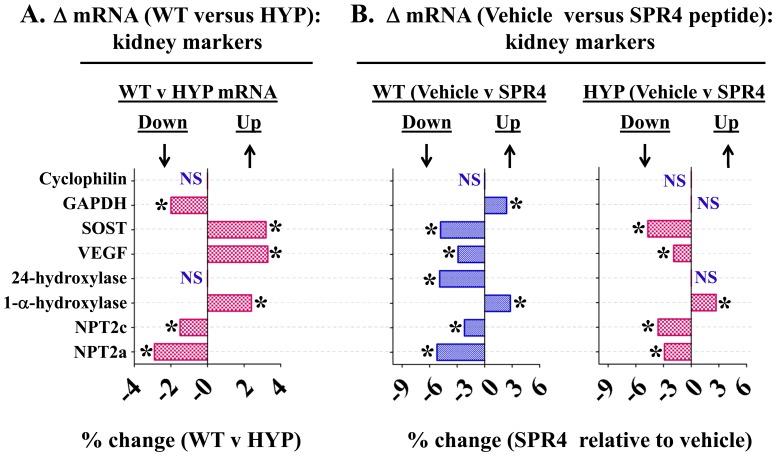
Whole kidney gene expression (mRNA) comparisons as measured by quantitative RT/PCR (qRT-PCR) for wild type (WT) and HYP mice infused with vehicle or SPR4 peptide for 28 days. Column headings represent; WT  =  wild type mice, HYP  =  X-linked hypophosphatemic rickets mice, SPR4  =  infused SPR4-peptide and Vehicle  =  Saline infused. For gene analysis mRNA was prepared from whole kidneys snap frozen in LN2 and homogenized. For qRT-PCR gene analysis fold differences in expression calculated by the Pfaffl method [163] were statistically analyzed for significance using the One Sample t-test and the Wilcoxon Signed rank-test with theoretical means set to 1. Results are significant (* = p<0.05) unless indicated by NS (see also **Table 3** for detailed statistics). ND =  Not done, NS  =  Not Significant***Index***: **Cyclophilin**  =  cyclophilin; **GAPDH**  =  Glyceraldehyde 3-phosphate dehydrogenase; **SOST**  =  Sclerostin; **VEGF**  =  Vascular Endothelial Growth factor; **24-Hydroxylase**  = 1,25-hydroxyvitamin D_3_ 24-hydroxylase (CYP24A1); **1-α-Hydroxylase**  =  25-hydroxyvitamin D_3_ 1-alpha-hydroxylase (CYP27B1); **NPT2c**  =  Sodium-dependent phosphate co-transporter (Slc34a3); **NPT2a**  =  Sodium-dependent phosphate co-transporter (Slc34a1); **NS**  =  not significant; * = P<0.05. Histogram bars to the left of zero on the axis indicate down regulation and to the right up regulation.

**Figure 4:**
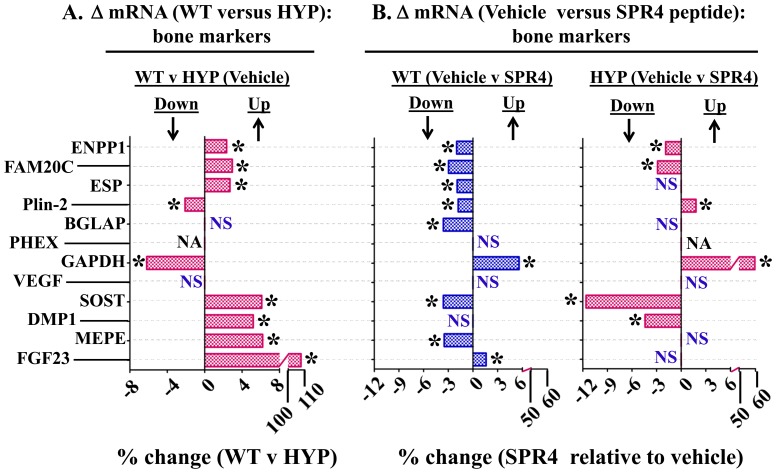
Bone (femur) gene expression (mRNA) comparisons as measured by quantitative RT/PCR (qRT-PCR) for wild type (WT) and HYP mice infused with vehicle or SPR4-peptide for 28 days. Mice were sacrificed on day 28 and femurs collected for RNA purification as described in methods. Column headings represent; WT  =  wild type mice, HYP  =  X-linked hypophosphatemic rickets mice, SPR4  =  infused SPR4-peptide and Vehicle  =  Saline infused. For gene analysis mRNA was prepared from bone marrow stromal cell “*depleted*” femurs as detailed in methods. For qRT-PCR gene analysis fold differences in expression calculated by the Pfaffl method [163] were statistically analyzed for significance using the One Sample t-test and the Wilcoxon Signed rank-test with theoretical means set to 1. Results are significant (* = p<0.05) unless indicated by NS (see also **Table 4** for detailed statistics). ***Index*** : **FAM20C**  =  Family with sequence similarity 20, member C Kinase also known as DMP4; **ENPP1**  =  Ectonucleotide Pyrophosphatase Phosphodiesterase 1; **ESP**  =  Osteotesticular protein tyrosine (OST-PTP); **Plin-2**  =  Perlipin-2; phosphatase; **Cyclophilin**  =  peptidylprolyl isomerase A (cyclophilin A); **BGLAP**  =  Osteocalcin or Bone Gamma-Carboxyglutamate (gla) protein; **PHEX**  =  Phosphate-regulating gene with Homologies to Endopeptidases on the X chromosome; **GAPDH**  =  Glyceraldehyde 3-phosphate dehydrogenase; **VEGF**  =  Vascular Endothelial Growth factor; **DMP1**  =  Dentin Matrix Protein 1; **SOST**  =  Sclerostin; **MEPE**  =  Matrix Extracellular Phosphoglycoprotein with ASARM -motif; **FGF23**  =  Fibroblast Growth Factor 23; **NS**  =  not significant; **NA**  =  not applicable, PHEX mutated in HYP; * = P<0.05. Histogram bars to the left of zero on the axis indicate down regulation and to the right up regulation.

**Figure 5:**
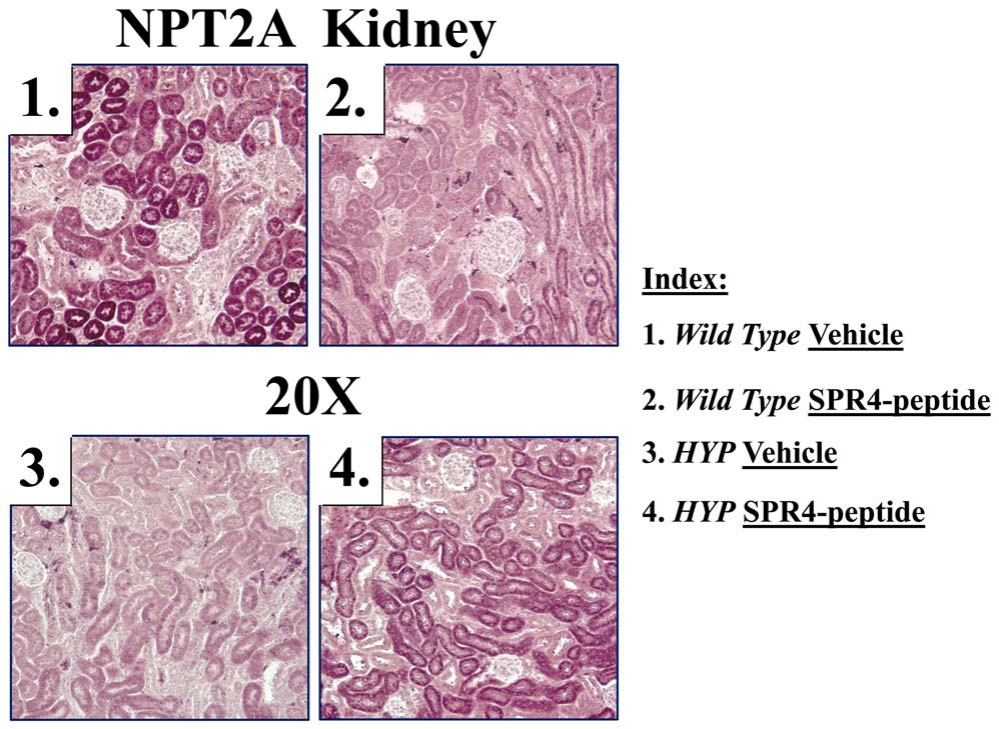
Immunohistochemistry of kidney sections confirm changes in protein expression for Na dependent phosphate co-transporter (NPT2A; Slc34a1). NPT2a protein-expression (purple-stain) in renal cortex sections is markedly decreased in HYP mice (compare photos 1 and 3). SPR4 peptide suppresses NPT2a expression in WT mice (compare photos 1 and 2) but increases NPT2a expression in HYP mice (compare photos 3 and 4). Staining is localized to proximal convoluted tubules with little glomerular staining. Magnifications are 20× and are from representative sections (matched regions).

HYP mice were hypophosphatemic with significantly increased circulating levels of FGF23, PTH, and ASARM-peptides. Of note, HYP mice also displayed increased circulating sclerostin (SOST), an inhibitor of the canonical Wnt/βCatenin pathway (Figures 1A, 2A and Tables 1, 2). Also consistent, an increase in renal and bone sclerostin (SOST) mRNA expression occurred in HYP mice relative to wild type (Figures 3A, 4A and Tables 3, 4). Intriguingly, as reported by other workers [76]–[79] HYP mice circulating 1.25(OH)_2_D_3_ remained inappropriately normal despite the hypophosphatemia (Figure 1A and Table 1) but HYP mRNA expression of renal 1-α hydroxylase was significantly increased (Figure 3A and Table 3). Also, consistent with previous reports (but counter intuitive to the increased FGF23), HYP renal mRNA expression of 24 renal hydroxylase was not significantly different to WT mice (Figure 3A and Table 3). Since FGF23 suppresses renal 1-α hydroxylase and induces 24 renal hydroxylase in WT mice, HYP mice likely have a post translational defect in Vitamin D metabolism [76]–[79]. The HYP mice hypophosphatemia was associated with a renal phosphate leak as deduced from an increased Fractional Excretion of Phosphate (FEP) (Figure 2A and Table 2). Also, as reported previously, a decreased renal expression of the Na^+^ dependent phosphate co transporters (NPT2a and NPT2c) occurred (Figures 3A, 5 and Table 3).

**Table 1:** Serum chemistry results and comparisons for wild type and HYP mice infused with vehicle or SPR4-peptide.

	WT			HYP			WT versus HYP
Serum Markers	VEHICLE (n = 5)	SPR4 (n = 5)	WT (% Change)	VEHICLE (n = 9)	SPR4 (n = 5)	HYP (% Change)	Vehicle (% Change)
			Vehicle ver SPR4			Vehicle ver SPR4	WT versus HYP
**Phosphorus mg/dL**	7.9±0.19	6.9±0.16^ab^	−12.66*	4.5±0.16^ac^	5.7±0.20^ab^	26.67*	−43.04*
**Calcium mg/dL**	9.1±0.25	10.4±0.26^a^	14.29*	9.4±0.30	9.6±0.40	**2.13**	**3.30**
**1,25(OH)2 pg/mL**	88.0±17.4	181.2±26.2^ab^	105.91*	112.0±8.1^c^	153.0±9.4^ab^	36.60*	**27.27**
**FGF23 pg/mL**	151.2±7.7	226.0±13.1^abc^	49.47*	2793.6±185^ac^	4620.6±553^ab^	65.39*	1747.62*
**PTH pg/mL**	42.9±7.7	91.8±12.4^abc^	113.99*	170.7±30.0^a^	148.9±12.9^a^	**−12.77**	297.9*
**Sclerostin ng/mL**	2.2±0.04	1.3±0.30^ab^	−40.91*	3.3±0.01^ac^	2.3±0.36^b^	−30.30*	50.00*
**ASARM nM**	60.45±2.6	ND	**ND**	92.2±4.5	ND	**ND**	52.52*
**Glucose mg/dL**	215.0±39.2	212.6±11.3^b^	**−1.12**	383.3 ±43.9^ac^	171.0±21.7^b^	−55.38*	78.28*
**Insulin pg/mL**	319.6±23.4	296.9±20.8^b^	**−7.1**	145.3±12.3^ac^	262.3±14.3^b^	80.52*	−54.54*
**Osteocalcin ng/mL**	23.6±1.6	16.1±2.2^a^	−31.78*	22.2±2.2^c^	33.0±2.5^ab^	48.65*	**−5.93**
**Leptin pg/mL**	429.4±86.2	171.0±41.1^a^	−60.18*	114.7±12.8^a^	147.3±40.0^a^	**28.42**	−73.29*
**Adiponectin υg/mL**	80.6±14.1	41.2±7.3^a^	−48.88*	48.2±4.3^a^	56.0±9.1^a^	**16.2**	−40.29*
**Adrenaline ng/mL**	0.53±0.02	0.46±0.04^a^	−15.2*	0.35±0.04^a^	ND	**ND**	−34.00*
**Uric Acid mg/dL**	5.4±0.31	3.5±0.68^a^	−54.3*	2.4±0.33^ac^	2.3±0.46^ab^	**−0.10**	−125.0*

Mice were sacrificed on day 28 and sera prepared from 16 hour fasted mice housed in metabolic cages. Column headings represent; WT  =  wild type mice, HYP  =  X-linked hypophosphatemic rickets mice, SPR4  =  infused SPR4-peptide and Vehicle  =  Saline infused. Values are means with ± SEM (N = 6). Unpaired t tests with confidence intervals of 95% were used for statistical analysis (see also **Figure 1**). Superscript letters (a, b or c) added to the calculated serum values indicate; a =  significantly different to WT vehicle (WT-VE) infused mice p<0.008, b =  significantly different to HYP vehicle (HYP-VE) infused mice p<0.002 and c =  significantly different to HYP SPR4-peptide (HYP-SPR4) infused mice p<0.02. ND =  Not done. The percentage changes between *WT-VE versus WT-SPR4*, *HYP-VE versus HYP-SPR4* and *WT-VE versus HYP-VE* are shown in columns 3, 6 and 7 as indicated in the headings. In these columns (3, 6 and 7), the numbers that are significantly different (p<0.01) are denoted by a superscript asterisk (*).

**Table 2:** Urine chemistry results and comparisons for wild type and HYP mice infused with vehicle or SPR4-peptide.

	WT			HYP			WT versus HYP
Urine Markers	VEHICLE (n = 5)	SPR4 (n = 5)	WT (% Change)	VEHICLE (n = 9)	SPR4 (n = 5)	HYP (% Change)	Vehicle (% Change)
			Vehicle ver SPR4			Vehicle ver SPR4	WT versus HYP
**FEP (%)**	8.7±1.33	18.3±2.2^ab^	110.34*	24.7±3.6^a^	15.4±3.6^a^	−37.65*	183.91*
**FE Ca (%)**	0.51±0.07	0.90±0.11^abc^	76.47*	0.5±0.08	0.40±0.1	**−20.0**	**−1.96**
**Calcium/Creatinine (mg/dL)**	0.10±0.01	0.18±0.02^a^	80.00*	0.15±0.01^a^	0.13±0.03	**−13.33**	50.00*
**ASARM/Creatinine (nM/mg dL-1)**	0.52±0.06	0.29±0.04^ab^	−44.23*	0.80±0.08^ac^	0.38±0.09^ab^	−52.5*	53.85*
**Creatinine Clearance (υL/min)**	50.8±10.17	30.69±4.5	**NS**	43.6±6.7	32.6±1.9	**−11.0**	**−3.41**
**Urine Creatinine (mg/15 hours)**	379.9±64.41	203.6±21.96^a^	−46.4*	182.7±27.19^a^	213.6±18.61^a^	**14.14**	−51.91*
**Fractional Excretion Uric Acid %**	6.8±0.7	9.0±1.4	32.2*	17.1±3.4	8.3±1.8	−106.0*	251.5*

Mice were sacrificed on day 28 and sera prepared from 16 hour fasted mice housed in metabolic cages. Column headings represent; WT  =  wild type mice, HYP  =  X-linked hypophosphatemic rickets mice, SPR4  =  infused SPR4-peptide and Vehicle  =  Saline infused. Values are means with ± SEM (N = 6). Unpaired t tests with confidence intervals of 95% were used for statistical analysis. Unpaired t tests with confidence intervals of 95% were used for statistical analysis (see also **Figure 2**). Superscript letters (a, b or c) added to the calculated serum values indicate; a =  significantly different to WT vehicle (WT-VE) infused mice p<0.008, b =  significantly different to HYP vehicle (HYP-VE) infused mice p<0.002 and c =  significantly different to HYP SPR4-peptide (HYP-SPR4) infused mice p<0.02. ND =  Not done. The percentage changes between *WT-VE versus WT-SPR4*, *HYP-VE versus HYP-SPR4* and *WT-VE versus HYP-VE* are shown in columns 3, 6 and 7 as indicated in the headings. In these columns (3, 6 and 7), the numbers that are significantly different (p<0.01) are denoted by a superscript asterisk (*). ***Index*** : **FEP(%)**  =  Fractional Excretion of Phosphate, **Fe Ca (%)**  =  Fractional Excretion of Calcium.

**Table 3:** Whole kidney gene expression (mRNA) as measured by quantitative RT/PCR (qRT-PCR) for wild type and HYP mice infused with vehicle or SPR4-peptide (see also **Figure 3**).

	Kidney					
mRNA	WT versus HYP		Vehicle versus SPR4			
	Vehicle		WT		HYP	
	Mean	Std Error	Mean	Std Error	Mean	Std Error
**NPT2a**	−2.9*	±0.25	−5.2*	±0.65	−2.9*	±0.67
**NPT2c**	−1.5*	±0.06	−2.2*	±0.29	−3.6*	±1.07
**1-α-Hydroxylase**	2.4*	±0.91	2.8*	±0.54	2.7*	±0.92
**24-Hydroxylase**	NS	**―**	−4.9*	±1.97	NS	**―**
**VEGF**	3.3*	±0.71	−2.9*	±0.65	−1.9*	±0.16
**SOST**	3.2*	±0.32	−4.8*	±1.1	−4.7*	±0.74
**GAPDH**	−2.0*	±0.28	2.4*	±0.89	NS	**―**
**Cyclophilin**	NS	**―**	NS	**―**	NS	**―**

Column headings represent; WT  =  wild type mice, HYP  =  X-linked hypophosphatemic rickets mice, SPR4  =  infused SPR4-peptide and Vehicle  =  Saline infused. For gene analysis mice were sacrificed on day 28 and mRNA prepared from whole kidneys as detailed in methods (N = 6). For qRT-PCR gene analysis fold differences in expression calculated by the Pfaffl method [163] were statistically analyzed for significance using the One Sample t-test and the Wilcoxon Signed rank-test with theoretical means set to 1. Results for all tests were considered to be significantly different at p<0.05 and are denoted with an asterisk (*) superscript. The fold changes in expression and standard error of the means (SEM) of *WT-VE versus HYP-VE*, *WT-VE versus WT-SPR4* and *HYP-VE versus HYP-SPR4* are shown in columns 1, 2 and 3 as indicated in the headings. ND =  Not done, NS  =  Not Significant. ***Index***: **NPT2a**  =  Sodium-dependent phosphate co-transporter (Slc34a1); **NPT2c**  =  Sodium-dependent phosphate co-transporter (Slc34a3); **1-α-Hydroxylase**  =  25-hydroxyvitamin D_3_ 1-alpha-hydroxylase (CYP27B1); **24-Hydroxylase**  = 1,25-hydroxyvitamin D_3_ 24-hydroxylase (CYP24A1); **VEGF**  =  Vascular Endothelial Growth factor; **SOST**  =  Sclerostin; **GAPDH**  =  Glyceraldehyde 3-phosphate dehydrogenase; **Cyclophilin**  =  peptidylprolyl isomerase A (cyclophilin A).

**Table 4:** Bone (femur) gene expression (mRNA) as measured by quantitative RT/PCR (qRT-PCR) for wild type and HYP mice infused with vehicle or SPR4-peptide (see also **Figure 4**).

	Bone					
mRNA	WT versus HYP		Vehicle versus SPR4			
	Vehicle		WT		HYP	
	Mean	Std Error	Mean	Std Error	Mean	Std Error
**FGF23**	107.7*	±30.39	1.6*	±0.25	NS	**―**
**MEPE**	6.2*	±1.41	−3.5*	±1.01	NS	**―**
**DMP1**	5.2*	±1.61	NS	**―**	−4.4*	±0.65
**SOST**	6.1*	±1.80	−3.6*	±0.96	−11.6*	±2.33
**VEGF**	NS	**―**	NS	**―**	NS	**―**
**GAPDH**	−6.2*	±1.72	5.6*	±1.02	59.7*	±17.91
**Osteocalcin**	NS	**―**	−3.6*	±0.76	NS	**―**
**Cyclophilin**	NS	**―**	NS	**―**	NS	**―**
**Plin-2**	−2.1*	±0.33	−1.8*	±0.25	1.8*	±0.22
**ESP**	2.7*	±0.81	−1.9*	±0.17	NS	**―**
**FAM20C**	2.9*	±0.30	−3.0*	±0.68	−2.9*	±0.59
**ENPP1**	2.3*	±0.36	−2.0*	±0.18	−1.9*	±0.20

Column headings represent; WT  =  wild type mice, HYP  =  X-linked hypophosphatemic rickets mice, SPR4  =  infused SPR4-peptide and Vehicle  =  Saline infused. For gene analysis mice were sacrificed on day 28 and mRNA prepared from bone marrow stromal cell depleted femurs as detailed in methods (N = 6). For qRT-PCR gene analysis fold differences in expression calculated by the Pfaffl method [163] were statistically analyzed for significance using the One Sample t-test and the Wilcoxon Signed rank-test with theoretical means set to 1. Results for all tests were considered to be significantly different at p<0.05 and are denoted with an asterisk (*) superscript. The fold changes in expression and standard error of the means (SEM) of *WT-VE versus HYP-VE*, *WT-VE versus WT-SPR4* and *HYP-VE versus HYP-SPR4* are shown in columns 1, 2 and 3 as indicated in the headings. ND =  Not done, NS  =  Not Significant. ***Index*** : **FGF23**  =  Fibroblast Growth Factor 23; **MEPE**  =  Matrix Extracellular Phosphoglycoprotein with ASARM -motif; **DMP1**  =  Dentin Matrix Protein 1; **SOST**  =  Sclerostin; **VEGF**  =  Vascular Endothelial Growth factor; **GAPDH**  =  Glyceraldehyde 3-phosphate dehydrogenase; **PHEX**  =  Phosphate-regulating gene with Homologies to Endopeptidases on the X chromosome; **Osteocalcin**  =  Bone gamma-carboxyglutamate (gla) protein; **Cyclophilin**  =  peptidylprolyl isomerase A (cyclophilin A); **Plin-2**  =  Perlipin-2; **ESP**  =  Osteotesticular protein tyrosine phosphatase (OST-PTP); **FAM20C**  =  Family with sequence similarity 20, member C Kinase also known as DMP4; **ENPP1**  =  Ectonucleotide Pyrophosphatase Phosphodiesterase 1.

### Energy metabolism, HYP versus WT: hyperglycemia, hypoinsulinemia, hypoleptinemia & hypouricemia

HYP mice were significantly hyperglycemic, hypoinsulinemic and hypouricemic (uric acid) with reduced circulating leptin and adiponectin levels. Also, circulating adrenaline a marker of sympathetic tone was significantly reduced in HYP mice (Figure 1A and Table 1). In contrast, circulating osteocalcin (BGLAP) and bone mRNA expression showed no significant difference between wild type and HYP mice (Figures 1A, 4A and Tables 1, 4). Of note, a significant decrease in bone (-6X) and renal (-2X) glyceraldehyde 3-phosphate dehydrogenase (GAPDH) mRNA expression occurred in HYP mice compared to WT mice (Figures 3A, 4A and Tables 3, 4). Thus, this gene (GAPDH) was assessed as unsuitable for use as a house keeping control with HYP mice. This was confirmed by cross referencing with cyclophilin and transferrin house-keeping genes (data not shown). Transferrin was therefore used as a house keeping gene for all quantitative RT/PCR experiments (see materials and methods). Since GAPDH is a glycolytic enzyme the reduction in bone-renal expression and hypoinsulinemia is consistent with the significant hyperglycemia measured in HYP mice. A significant increase in HYP vascular endothelial growth factor (VEGF) mRNA occurred in kidney with no change in bone (Figures 3A, 4A and Tables 3, 4). VEGF is a vasculogenic and angiogenic factor with abnormal expression in diabetes [80], [81]. A marked increased expression of two genes recently associated with two newly characterized inherited forms of autosomal hypophosphatemic rickets occurred in HYP mice bone. Specifically, increased FAM20C a bone ASARM-motif specific kinase) [72]–[75] and Ectonucleotide Pyrophosphatase Phosphodiesterase 1 (ENPP1) [82]–[84] occurred (Figure 4A and Table 4). Increased bone expression of osteotesticular protein tyrosine phosphatase (ESP or OST-PTP) also occurred with HYP mice consistent with the observed hyperglycemia and abnormal insulin/leptin levels (Figure 4A and Table 4). Specifically, both ESP and ENPP1 mutations and over expression are associated with abnormalities in glucose metabolism, insulin sensitivity, obesity and diabetes [85]–[89].

Because of the association of uric acid with sugar metabolism, serum and urine uric-acid levels were measured. A marked and significant hypouricemia (−125%) occurred with HYP-VE mice relative to WT-VE mice (Figure 1A and Table 1). This was accompanied by an increased urinary fractional excretion of uric acid (FE_UA) (Figure 2A and Table 2) that suggests an abnormality in HYP-VE mice uric acid renal-handling. The hypouricemia is also consistent with the hyperglycemia and observed changes in insulin, leptin and adiponectin. In summary, HYP mice showed significant defects in glucose and fat metabolism markers

### SPR4-peptide alters serum/urine chemistry and suppresses sclerostin (SOST) in WT & HYP mice

SPR4 peptide infusion induced hypophosphatemia in WT mice (WT-SPR4) but in contrast caused a marked 27% increase in serum phosphorus in HYP mice (HYP-SPR4) (Figure 1B; Table 1). This was reflected by a 100% increase in WT-SPR4 mice fractional excretion of phosphate (FEP) and a contrasting 38% decrease in HYP-SPR4 mice FEP (Figure 2B; Table 2). FGF23 levels increased by 50% in WT mice infused with SPR4 (Figure 1B; Table 1) and this was accompanied by a significant 1.6 fold increase in bone FGF23 mRNA expression (Figure 4B; Table 4). In contrast, HYP mice SPR4-treatment did not affect FGF23 mRNA expression (Figure 4B and Table 4) but circulating FGF23 was increased on a background of markedly elevated FGF23 (Figure 1B and Table 1). This was despite the SPR4 induced improvement in HYP mice serum phosphorus. Intriguingly, a similar FGF23 mRNA expression (decrease) and full length FGF23 protein expression (increase) occurs with HYP mice treated with an inhibitor of a proprotein-convertase (SPC2) coactivator protein, 7B2 [77]. Of note, SPR4 treated HYP mice serum PTH was decreased but this did not reach statistical significance (Figure 1B; Table 1). In contrast to HYP-SPR4 treated mice, WT-SPR4 mice exhibited a significant increase in serum PTH (114%) relative to WT vehicle mice, (Figure 1B; Table 1). A marked and significant suppression of circulating sclerostin (WT-SPR4  = −40% HYP-SPR4  = −30%), bone mRNA expressed sclerostin (WT-SPR4  = −3.6; HYP-SPR4  = −11.6) and renal mRNA expressed sclerostin (WT-SPR4  = −4.8; HYP-SPR4  = −4.7) occurred with both WT and HYP mice treated with SPR4-peptide (Figures 1B, 3B & 4B and Tables 1, 3 and 4).

### Corrected Energy metabolism (glucose, insulin, osteocalcin, ENPP1 & FAM20C), SPR4 treated HYP mice

The significant and marked HYP-mice fasting hyperglycemia and hypoinsulinemia were corrected by infusion with SPR4 peptide (Figure 1A & B; Table 1). Specifically, there were no significant differences between HYP SPR4 treated mice and WT vehicle mice with glucose and insulin serum metrics (Table 1). SPR4 peptide induced a major and significant reduction (−60%) in WT type but not HYP serum leptin. Notably, a non-significant trend towards an increase in serum leptin with HYP SPR4 treated mice occurred (Figure 1B and Table 1). Indeed, vehicle and SPR4-treated “HYP-mice” leptin serum levels remained dramatically reduced (−73%) compared to WT vehicle mice (Table 1). Intriguingly circulating adiponectin levels showed the same pattern with a significant reduction in SPR4 treated WT mice but not SPR4 treated HYP mice (Table 1). Sympathetic tone as represented by circulating adrenaline was significantly reduced in WT mice treated with SPR4 (Figure 1B and Table 1). Circulating and bone osteocalcin (BGLAP) levels of HYP and WT vehicle mice were not significantly different. In contrast, SPR4 peptide treatment induced a pronounced and significant increase (+48%) in HYP mice circulating osteocalcin that was not reflected by increased bone mRNA expression (Figures 1B, 4B and Tables 1, 4). Thus, more studies are required to determine whether the transcription independent SPR4 induced changes in HYP mice osteocalcin circulating levels are because of increased mRNA stability, decreased proteolysis or changes in post translational processing. Of note, the ELISA we used to measure circulating osteocalcin detects both inactive “γ-carboxylated” and active “γ-decarboxylated” forms. It is therefore possible that SPR4 treatment also induces a dynamic shift in the levels of *active* unbound osteocalcin (γ-decarboxylated) and *inactive* bound osteocalcin (γ-carboxylated osteocalcin has high affinity for hydroxyapaptite). In contrast to HYP mice, SPR4 peptide induced a major and significant reduction of both circulating osteocalcin and mRNA osteocalcin (BGLAP) expression in wild type mice bone (Figures 1B, 4B and Tables 1, 4). SPR4 treatment of both WT and HYP mice caused a striking and significant increase in bone and kidney GAPDH expression. Specifically, SPR4 treated mice induced a significant ×60 fold and ×6 fold increases in GAPDH bone expression for HYP and WT mice respectively (Figure 4B; Table 4) and a ×2.5 fold increase in WT kidney (Figure 3B; Table 3). For both HYP and WT mice, SPR4 treatment suppressed VEGF renal mRNA expression by ×2 and ×3 fold respectively with no significant differences in bone VEGF expression (Figures 3B, 4B and Tables 3, 4). SPR4 infusion also reduced bone expression of both ENPP1 and ESP in WT mice and suppressed ENPP1 in HYP mice (Figure 4B and Table 4). A marked and significant reduced expression of bone FAM20C mRNA occurred with both HYP and WT mice treated with SPR4-peptide (Figure 4B and Table 4). Thus the abnormal increased expression of FAM20C and ENPP1 observed in HYP-VE mice relative to WT-VE mice was reversed by SPR4-treatment.

SPR4 treatment induced a significant and marked decrease in WT serum uric acid levels (Figure 1B and Table 1). This was mirrored by an increase in the fractional excretion of uric acid (Figure 2B and Table 2). Although SPR4 induced a marked and significant change with WT-SPR4 mice uric acid levels, the hypouricemia present in HYP-VE mice was not corrected or exacerbated by SPR4 treatment (Figures 1A, 1B and Table 1). Also of note, although SPR4 did not induce any measureable effects on HYP mice hypouricemia (Figures 1B and Table 1) the SPR4 treatment did significantly reduce HYP-mice fractional excretion of uric acid (Figures 2B and Table 2).

### Fat Mass (DEXA) vehicle treated: HYP mice have markedly reduced fat mass and fat/mass ratios

**Figure 6:**
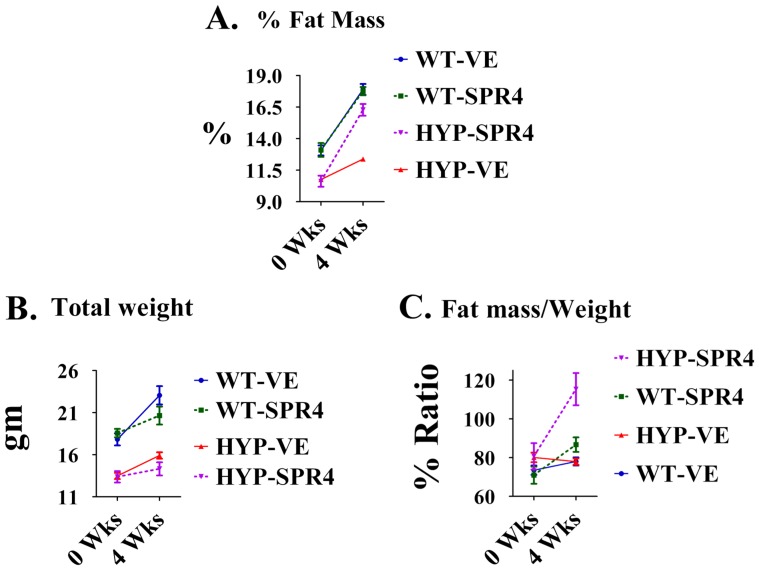
SPR4-peptide induces a dramatic increase in fat mass/weight in HYP and WT mice. Dual Energy X-ray Absorptiometry (DEXA) measurements using a Lunar PIXImus system were carried out as described previously and discussed in methods [140]. Measurements are shown for mice prior to pump implantation and after sacrifice 28 days later. The temporal percentage change measurements are shown in Figure 7 and Table 5. (**A**) **Percentage Fat Mass (% FAT Mass)**. HYPVE %-Fat-Mass was significantly less than WTVE %-Fat-Mass at all time-points. SPR4 peptide treatment significantly increased time-dependent gain in %-Fat-Mass for HYP mice (HYPSPR4) but not WT mice (WTSPR4) relative to respective vehicle groups. Following 2-way ANOVA analysis, phenotypic variation (including SPR4-treatment) was highly significant accounting for 31.86% of the total variance (F = 46.72, DF_n_ = 3, Df_d_ = 32 and *P<0.0001*). Also, time-changes were highly significant accounting for 53.69% of the total variance (F = 236.22, DF_n_ = 1, Df_d_ = 32 and *P<0.0001*). The phenotype/time *interaction* was also significant accounting for 7.18% of the total variance (F = 10.52, DF_n_ = 3, Df_d_ = 32 and *P<0.0001*). (**B**) **Total Weight (gm).** HYPVE-mice weight was significantly less than WTVE-mice weight at all time-points. SPR4 peptide treatment significantly decreased time-dependent gain in weight for both HYP mice (HYPSPR4) and WT mice (WTSPR4) relative to respective vehicle groups. Following 2-way ANOVA analysis, phenotypic variation (including SPR4-treatment) was highly significant accounting for 64.65% of the total variance (F = 40.6, DF_n_ = 3, Df_d_ = 32 and *P<0.0001*). Also, time-changes were highly significant accounting for 13.64% of the total variance (F = 25.69, DF_n_ = 1, Df_d_ = 32 and *P<0.0001*). The phenotype/time *interaction* was significant accounting for 4.73% of the total variance (F = 2.97, DF_n_ = 3, Df_d_ = 32 and *P = 0.0463*). (**C**) **Ratio of Fat mass/Weight (% Ratio).** No significant differences in fat-mass/weight ratios were observed between groups at 0 weeks (baseline, prior to pump implantation). In contrast, SPR4 peptide treatment significantly increased time-dependent gain in fat-Mass/weight ratio for both HYP mice (HYP-SPR4) and WT mice (WTSPR4) relative to respective vehicle groups. The gain in HYP-SPR4 fat-Mass/weight ratio was more marked and significantly greater than the WT-SPR4 mice. Following 2-way ANOVA analysis, phenotypic variation (including SPR4-treatment) was highly significant accounting for 30.60% of the total variance (F = 9.86, DF_n_ = 3, Df_d_ = 32 and *P<0.0001*). Also, time-changes were highly significant accounting for 17.14% of the total variance (F = 16.58, DF_n_ = 1, Df_d_ = 32 and *P = 0.0003*). The phenotype/time *interaction* was significant accounting for 19.17% of the total variance (F = 6.18, DF_n_ = 3, Df_d_ = 32 and *P = 0.002*). ***Index*** : **WTVE** =  wild type mice infused with vehicle (0.9% physiological saline); **HYPVE**  =  X-linked hypophosphatemic rickets mice infused with vehicle (0.9% physiological saline); **WTSPR4**  =  wild type mice infused SPR4-peptide; **HYPSPR4**  =  X-linked hypophosphatemic rickets mice infused SPR4-peptide.

**Figure 7:**
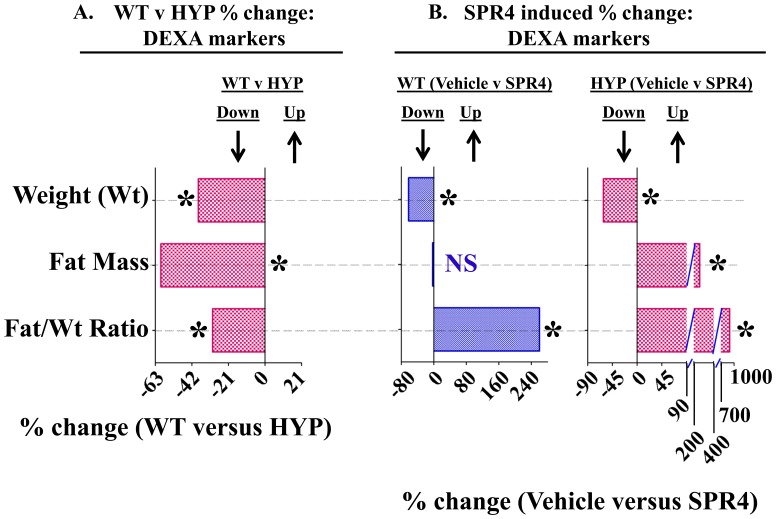
Temporal changes in (Weight, fat mass and fat mass/weight ratio) as measured by Dual Energy X-ray Absorptiometry (DEXA) for wild type and HYP mice infused with vehicle or SPR4-peptide for 28 days (See **Figure 6** for static changes). The mean percentage change over the 28 days for each metric was calculated and the percentage differences between the groups plotted as a histogram (see also **Table 5**). Values are mean percentage differences and are significant (* = P<0.05) unless indicated by NS (unpaired t test confidence interval  = 95%). Column headings represent; **WT**  =  wild type mice, **HYP**  =  X-linked hypophosphatemic rickets mice, **SPR4**  =  infused SPR4-peptide and **Vehicle**  =  Saline infused. Histogram bars to the left of zero on the y-axis indicate down regulation and to the right up regulation. DEXA measurements using a Lunar PIXImus system were carried out as described previously and discussed in methods [140]. ***Index*** : **Weight (Wt)**  =  percentage difference in weight change over 4 weeks; **Fat-Mass**  =  percentage difference in total fat-mass change over 4 weeks; **Fat/Wt Ratio**  =  percentage difference in total fat-mass/weight change over 4 weeks.

A decrease in overall fat mass of HYP vehicle mice relative to WT vehicle mice occurred at all time-points (Figure 6A). When corrected for weight (Figure 6B), the HYP-VE mice fat-mass/weight ratio was not significantly different to WT-VE mice at the start or after 4 weeks (Figure 6C). This was contrasted by significant decreases in time-dependent, dynamic measurements of fat-mass, fat-mass/weight ratios and weight that occurred with HYP vehicle mice compared to WT vehicle mice over 4 weeks (Figure 7A and Table 5). Intriguingly, a significant reduction in perilipin-2 (Plin-2) bone expression occurred with HYP-VE mice (Figure 4A and Table 4). Plin-2 is expressed in multiple non-adipose tissues and is thought to play a role in regulating lipid storage properties. Since Plin-2 null mice are protected against diet induced obesity[90], the decreased fat mass in HYP-VE mice is consistent with the reduced expression of Plin-2. There were no significant differences in feeding rates between any of the mice groups in the study (data not shown).

**Table 5:** Temporal changes in (Fat-Mass and Weight) as measured by Dual Energy X-ray Absorptiometry (DEXA) for wild type and HYP mice infused with vehicle or SPR4-peptide (See **Figure 7** for static changes).

	WT			HYP			WT & HYP
	VEHICLE (n = 5)	SPR4 (n = 5)	WT (% Change)	VEHICLE (n = 9)	SPR4 (n = 5)	HYP (% Change)	Vehicle (% Change)
DEXA			Vehicle ver SPR4			Vehicle ver SPR4	WT ver HYP
**%Δ-FAT/%Δ-Wgt**	1.33±0.14	4.8±1.01^abc^	260.1*	0.93±0.18^a^	9.31±2.5^ab^	901.1*	−30.1*
**% Change in Fat Mass (%Δ-FAT)**	37.5±5.0	36.4±5.3^bc^	**−2.93**	15.1±1.6^ac^	54.3±7.4^ab^	259.6*	−59.8*
**% Change in Weight (%Δ-Wgt)**	29.5±4.6	11.2±5.0^a^	−62.03*	18.2±2.9^ac^	7.0±1.3^ab^	−61.5*	−38.3*

DEXA measurements using a Lunar PIXImus system were carried out as described previously and discussed in methods [140]. Values are means (± SEM) of percentage changes that occurred from the start of the experiment (day 1; prior to pump implantation) to the end of the experiment (28 days later). Specifically, two measurements were taken, one on day 1 (baseline) and one on day 28. Mice were sacrificed on day 28 (age 9 weeks at sacrifice). Column headings represent; WT  =  wild type mice, HYP  =  X-linked hypophosphatemic rickets mice, SPR4  =  infused SPR4-peptide and Vehicle  =  Saline infused. Unpaired t tests with confidence intervals of 95% were used for statistical analysis. Superscript letters (a, b or c) added to the calculated values indicate; a =  significantly different to WT vehicle (WT-VE) infused mice p<0.008, b =  significantly different to HYP vehicle (HYP-VE) infused mice p<0.002 and c =  significantly different to HYP SPR4-peptide (HYP-SPR4) infused mice p<0.02. ND =  Not done. The percentage changes between *WT-VE versus WT-SPR4*, *HYP-VE versus HYP-SPR4* and *WT-VE versus HYP-VE* are shown in columns 3, 6 and 7 as indicated in the headings. In these columns (3, 6 and 7), the numbers that are significantly different (p<0.01) are denoted by a superscript asterisk (*). ***Index*** : **%Δ-Fat**  =  percentage change in fat-mass over 4 weeks; **%Δ-Wgt**  =  percentage change in weight over 4 weeks; **%Δ-Fat/%Δ-Wgt**  =  ratio of the changes in fat-mass and weight over 4 weeks.

### Fat Mass (DEXA): SPR4 infusion of HYP and Wild Type mice increases the fat mass/weight ratio

As stated earlier, there were no significant differences in food intake between all mice groups (data not shown). However, SPR4-treatment significantly reduced weight gain in HYP and WT mice treated with SPR4 (Figure 6B and 7B; Table 5). Notably, SPR4-treatment induced a marked and significant increase in % Fat Mass for HYP mice but not WT treated mice (Figure 6A). When corrected for weight, SPR4 treatment induced a significant increase in the “fat-mass/weight” ratio for both WT (WT-SPR4) and HYP (HYP-SPR4) treated mice (Figure 6C). The SPR4-peptide induced static changes were mirrored by significant dynamic/temporal increases in fat -mass/weight over the 4 weeks of infusion (Figure 7B; Table 5). Also, the induced static and temporal increases were significantly greater with HYP-SPR4 mice compared to WT-SPR4 mice. Of note, a suppressed dynamic and static fat-mass and fat-mass/weight ratio occurred with HYP-VE mice compared to WT-VE mice and this was accompanied by a marked suppression of bone Plin-2 gene-expression (Figures 4A, 6A, 6C, 7A and Tables 4 and 5). In contrast, SPR4-treatment had an opposite effect on HYP-mice. Specifically, HYP-SPR4 mice had a significant increase in Plin2 expression (Figure 4B and Table 4) and an increase in static and dynamic % fat-mass/weight (Figures 6A, 6C and 7B; Table 5). Remarkably, SPR4 treatment of WT mice (WT-SPR4) reduced Plin2 expression mimicking the HYP phenotype (Figure 4B and Table 5) but these mice also exhibited a significant increase in static and dynamic % fat-mass/weight ratio relative to WT-VE mice (Figures 6C and 7B; Table 5). Since Plin-2 null mice are protected against diet induced obesity [90], these changes indicate SPR4 may influence fat-mass by; (1) inhibiting PHEX activity in WT mice and/or (2) sequestering and neutralizing excess ASARM-peptides in PHEX defective HYP-mice.

## Discussion

Although phosphate levels are not the sole mediator of mineralization defects in familial hypophosphatemic disorders it is well documented that hypophosphatemia or systemic phosphate status correlates with changes in glucose production, energy metabolism and oxygen consumption [50], [53], [91]–[99]. More recently, the familial hypophosphatemic rickets disorders all show changes in glucose, insulin sensitivity and fat metabolism [50]–[53], [92]–[94], [100]–[102]. Hypophosphatemia is also associated with metabolic syndrome and because phosphate is involved in carbohydrate metabolism low serum phosphate compromises utilization of glucose, increases insulin resistance and induces hyperinsulinemia [96], [98], [103]. Also, patients with primary hyperparathyroidism have impaired glucose-tolerance, hyperglycemia and reduced insulin sensitivity [104]. Our studies and others show HYP mice have increased serum PTH with hyperglycemia and hypoinsulinemia [50]–[53], [102]. Also, consistent with the HYP mice hypoinsulinemia and hyperglycemia a complete loss of insulin with hyperglycemia in diabetes type 1 patients (DM1) is associated with a loss of bone mineral density [105]. Of relevance, a recent microarray study showed major up-regulation of genes belonging to the PPAR-γ family (notably adiponectin, a marker of insulin resistance [106]) and PHEX during mineralization of osteoblast cultures over 27 days [107]. Adiponectin stimulates the proliferation, differentiation, and mineralization of osteoblasts via the AdipoR1 and AMP kinase signaling pathways in autocrine and/or paracrine fashions [108]. Thus the down regulation of adiponectin in HYP-mice shown in this study may also contribute to the abnormal bone phenotype. Moreover, there is an association of low serum phosphate levels with glucose intolerance, insulin sensitivity and insulin secretion in non-diabetic healthy-subjects [95] and a phosphate deplete diet impairs rat insulin secretion (markedly reduced) by pancreatic islets *ex vivo* [109]. HYP mice also have increased hepatic glucose-6-phosphatase activity [53] and rats fed a phosphate deplete diet up-regulate expression and activity of this enzyme [97], [110]. Also, overexpression of glucose-6-phosphatase in rats induces glucose intolerance, hyperglycemia with changes in circulating free fatty acids and triglycerides [111]. Remarkably, targeted deletion of the renal proximal-tubule insulin-receptor in mice promotes hyperglycemia, up regulation of glucose 6 phosphatase and gluconeogenesis [112]. This is of interest since the renal proximal tubule contains the Na-dependent phosphate cotransporters (NPT2a and NPT2c) and hypophosphatemia negatively regulates insulin synthesis and sensitivity [53], [95], [96], [98], [103], [109], [113], [114]. Although the liver is traditionally thought to be the major organ involved in glucose homeostasis the kidney is now also well recognized as a major player [115]–[117]. There are also strong correlations with FGF23, obesity and insulin resistance [118], [119]. Indeed, cardiovascular disease (CVD) and non-insulin-dependent diabetes mellitus (NIDDM) in obese patients have been proposed to be directly caused by hypophosphatemia [113]. Specifically, low serum phosphate adversely affects glucose metabolism resulting in hyperglycemia, with increased risk of NIDDM, hypertension and increased risk of stroke [113]. In obese individuals, a major role for phosphate in regulatory thermogenesis and dysregulation of the basal metabolic rate occurs [113], [120]. Also, alterations in red cell glycolytic intermediates and oxygen transport due to a striking increase in red cell oxygen affinity occur in hypophosphatemic subjects [121]. These changes are accompanied by defective ATP synthesis and reduced renal cortical ribonucleoside triphosphate pools [122], [123]. The alterations in oxygen-affinity and glucose-metabolism in hypophosphatemic subjects is due primarily to regulatory abnormalities at the glyceraldehyde-3-phosphate dehydrogenase (GAPDH) step [121]. In line with this, defective ATP synthesis and impaired thermoregulatory regulation with increased metabolic rate and oxygen consumption also occurs with X-linked hypophosphatemic rickets mice (HYP) [49], [123]. Also, our study shows that there is a major reduction in HYP renal and bone GAPDH expression.

To investigate the underlying cause for the changes in glucose metabolism in HYP mice we measured expression of key genes known to play a bone-renal role in energy metabolism. The control of glucose metabolism in mice involves regulation of osteocalcin activity through a bimodal mechanism [85], [124]: (1) osteocalcin activity is regulated negatively by ESP, the gene for osteotesticular protein tyrosine phosphatase (OST-PTP). OST-PTP (ESP) dephosphorylates and inactivates the osteoblast insulin receptor that results in increased γ-carboxylation of osteocalcin and reduced osteocalcin bioactivity [85]. This then leads to hypoinsulinemia, reduced insulin sensitivity, hyperglycemia and glucose intolerance [85] and; (2) bone resorption reduces osteoclastic pH and increases acidity that then spontaneously decarboxylates and activates osteocalcin [124]. This results in increased insulin sensitivity and improved glucose tolerance. Respective to mechanism 2 we do not have a serum assay that differentiates between γ-carboxylated or decarboxylated osteocalcin. However, although there were no changes in serum osteocalcin with HYPVE mice we found decreased serum osteocalcin with WT-SPR4 mice and increased serum osteocalcin with HYP-SPR4 mice. With WT-SPR4 treated mice, a reduction in serum uric acid, sympathetic tone, adiponectin, leptin and serum phosphate with increased FGF23 and PTH occurred, mimicking the changes in HYP-VE mice. However, in contrast to HYPVE mice, WT-SPR4 mice had reduced osteocalcin and sclerostin with markedly increased 1.25(OH)_2_D_3_ and hypercalcemia. Of note, 1.25(OH)_2_D_3_ is reported to inhibit the deleterious effects of high glucose on osteoblasts through undercarboxylated osteocalcin and insulin signaling [100], [125]. Thus with WT mice treated with SPR4-peptide (binds to and inactivates ASARM-motif and peptide), the dramatic increase in 1.25(OH)_2_D_3_, hypercalcemia and reduced sclerostin may have helped to counteract the reduced osteocalcin resulting in normal glucose and insulin levels. Also, from these findings it is possible that despite decreased osteocalcin expression in WT-SPR4 mice, an increase in decarboxylated, active-osteocalcin occurred. Indeed, others have shown the ratio of undercarboxylated to total osteocalcin is a more reliable marker of osteocalcin activity than the total level of undercarboxylated osteocalcin [126]. In contrast, with HYP-VE mice, osteocalcin levels remained unchanged and the abnormal bone turnover likely contributed to an increase in the γ-carboxylated/decarboxylated osteocalcin ratio and thus reduced active osteocalcin. In further support of this model, HYP-VE mice showed increased ESP bone expression, the gene coding for osteotesticular protein tyrosine phosphatase (OST-PTP). ESP or OST-PTP phosphatase dephosphorylates and inactivates the osteoblast insulin receptor and inhibits the metabolic function of osteocalcin, resulting in reduced insulin secretion, hyperglycemia and reduced glucose tolerance [124]. Also, HYP-VE bone Ectonucleotide-Pyrophosphatase-Phosphodiesterase-1 (ENPP1) was markedly increased relative to WT-VE mice. ENPP1 mutations cause autosomal recessive hypophosphatemic rickets (ARHR2; MIM 173335) in mice and man [82], [83], [127]–[129] and over expression induces hyperglycemia, insulin resistance and diabetes [86]–[89], [130]–[134]. Like ESP, ENPP1 is an inhibitor of insulin signaling. It does this by interacting with the insulin receptor (IR) and decreasing IR β-subunit auto-phosphorylation [87], [133], [134]. Thus, our observed increased expression of both these genes (ENPP1 and ESP) is consistent with the HYP mice hyperglycemia and altered insulin sensitivity.

A marked and significant increased bone-expression of FAM20C kinase occurred in HYP mice relative to WT-mice. Of relevance, targeted deletion of FAM20C in mice results in autosomal hypophosphatemic rickets (ARHR 2) [72]–[75]. Recent research has shown this kinase specifically phosphorylates ASARM-motifs derived from SIBLING proteins (MEPE, DMP1, osteopontin etc.) [75], [135]. This is consistent with previous studies that show phosphorylation of the ASARM-motif is important for specific interaction, binding and substrate hydrolysis by PHEX, a Zn-metalloendopeptidase responsible for X-linked hypophosphatemic rickets [55], [56], [59]–[61], [66]. Notably, a lack of phosphorylation of this motif would be expected to result in impaired [(PHEX)-(DMP1-ASARM)-(α_5_β_3_-integrin)] binding and thus increased FGF23 mRNA expression and circulating FGF23 protein (as observed in ARHR2 and also HYP mice). Recent elegant studies suggest that phosphorylation of FGF23 by FAM20C also occurs and this renders FGF23 resistant to O-glycosylation [136]. This is important because O-glycosylation protects FGF23 from furin mediated proteolytic cleavage and increases half-life of circulating full-length FGF23 [137]. Consistent with this, mutations in N-acetylgalactosaminyltransferase (GalNac-T3) that cause impaired O-glycosylation results in tumoral calcinosis and hyperphosphatemia due to reduced half-life of FGF23 [137]. Thus, mutations in FAM20C (ARHR2) leading to reduced FGF23 phosphorylation and thereby increased O-glycosylation may also play a role in maintaining increased levels of furin resistant full-length active FGF23. In overview, phosphorylation of ASARM by FAM20C is likely responsible for the observed reduced expression and protein production of FGF23, also, FAM20C phosphorylation of FGF23 is likely responsible for the reduced stability and half-life of FGF23. As mentioned earlier, in our HYP mice we observed over expression of FAM20C. This suggests an attempted FAM20C feedback mechanism in PHEX defective HYP-mice designed to correct the massively increased expression and production of FGF23. Unfortunately, the vastly increased FGF23 production in HYP mice (Wild Type mice; 50 pg/mL and HYP mice; 5000 pg/mL) clearly overwhelms the inhibitory effects (i.e. reduced half-life of FGF23) induced by the increased HYP-mice FAM20C levels. Of interest, infusion of SPR4-peptide in both WT and HYP mice results in a marked suppression of bone FAM20C kinase. Since these SPR4-treated mice have improved energy metabolism this suggests a possible FAM20C, ATP, phosphate regulation and mineral metabolism nexus.

As discussed earlier, HYP mice have increased oxygen consumption and metabolic rate consistent with a defect in aerobic metabolism [49]. The increased oxygen consumption is not associated with abnormal thyroid hormone T4 levels, altered body weight or surface to weight ratios. Also, the altered oxygen consumption is reportedly independent of the hypophosphatemia [49]. Strikingly, HYP mice have a marked increase in cardiac output (blood flow) to liver, muscle and bone. Of direct relevance, oxygen tension is an important mediator of the transformation of osteoblasts to osteocytes [138]. Osteocytes are predominantly exposed to low partial pressures of oxygen (pO_2_) levels within the embedded lacuno-canalicular complex of bone [139]. A recent study used an osteocyte cell line MC3T3-E1 to investigate the effects of hypoxia in cell culture and showed major differences in the expression of osteocyte expressed proteins DMP1, MEPE, FGF23 and Cx43 [138]. Also, changes in osteocalcin and alkaline phosphatase occurred under hypoxic conditions and this positively influenced osteoblast to osteocyte transformation [138]. Thus, the increased blood flow, increased oxygen-uptake and metabolism may play a key role in the bone-mineralization and energy metabolism abnormalities in HYP-mice. Of direct relevance, our earlier studies using BaSO_4_ perfusion coupled with μCT showed altered vasculature in MEPE transgenic mice[140]. Specifically, increased blood vessel number occurred in the kidneys and bones that was accompanied by increased expression of VEGF [140]. The MEPE-tgn mice like HYP mice have increased circulating and urinary ASARM-epitopes as well as MEPE expression [7], [54], [64], [140], [141].

Since GAPDH and defective ATP synthesis are implicated in the abnormal an/aerobic and glucose metabolism found in hypophosphatemia [49], [121]–[123], we measured expression of glyceraldehyd-3-phosphate dehydrogenase (GAPDH) in bone and kidney. GAPDH is a key enzyme of the glycolytic pathway and sits at the portal of the first and second stages of glucose catabolism. The first stage of glycolysis consumes 2 molecules of ATP and is thus energy expensive. The second stage generates 4 molecules of ATP, a net energy gain of 2 molecules of ATP. Of relevance, insulin tightly regulates GAPDH activity and the GAPDH gene contains a positive insulin response element (IRE) [142], [143]. Consistent with the reduced insulin, a major suppression of GAPDH occurs in bone and kidneys of HYP-VE mice. The GAPDH gene is responsible for the conversion of glyceraldehyde-3-PO4 (G3P) to 3-phosphoglyceroyl phosphate (1,3-BPG), the final step of stage 1 of glycolysis (energy expensive stage). As discussed, stage 2 of glycolysis begins by the conversion of 1,3-BPG to 3-phosphoglycerate and generates 2 molecules of ATP and this in turn leads to the generation of another 2 molecules of ATP downstream (a net gain of 2 ATPs). The suppression of GAPDH is therefore expected to “bottleneck” stage 1 of glycolysis and reduce flux through stage 2. This in turn will negatively impact the generation of ATP, NADH and acetyl-CoA, the terminal intermediate of glycolysis. Acetyl-CoA links glycolysis to the tricarboxylic acid cycle (TCA) and so a reduction of this intermediate will reduce further the availability of NADH for mitochondrial oxidative-phosphorylation. Thus, suppressed GAPDH activity in the HYP-VE mice is expected to severely compromise the efficiency of glucose catabolism via glycolysis, TCA and oxidative phosphorylation.

Our experiments provide compelling evidence for how the above changes in HYP-mice induce a metabolic adaptive response that compensates for the impaired anaerobic glucose catabolism. Specifically, the TCA cycle is fed not only by glucose breakdown (glycolysis) but also by fatty acid degradation and amino acid catabolism. Also to be considered is the redirection of glyceraldehyde-3-PO4 degradation to the pentose phosphate pathway (PPP) and purine degradation that in turn impacts uric acid production. Of relevance, the PPP pathway is reportedly severely impaired in HYP mice[50], [123] with reduced ribonucleoside triphosphate pools [123]. Also, increased gluconeogenesis occurs in HYP-mice bone, kidney and liver [50], [51], [53], [102], [144]. Notably a key gluconeogenic enzyme glucose-6-phosphatase is up regulated in livers of HYP-mice resulting in hyperglycemia [53] and as discussed our studies show down regulation of GAPDH a key glycolytic enzyme. The fact that the suppressed PPP pathway and the increased gluconeogenesis are evident *in vivo* and *in vitro* (bone and kidney cell cultures) suggests intrinsic defects such as increased acidic ASARM-peptides may play a role. In support of this the substrate profile for the increased HYP glucose production is similar to that described for the stimulatory effects of cAMP [145] and acidic pH [52], [146]. Also, the HYP-mice hypoinsulinemia measured by us and others is consistent with the observed hyperglycemia and increased gluconeogenesis particularly given the regulatory link with hypophosphatemia and insulin secretion and sensitivity [53], [95], [96], [98], [103], [109], [113]. Of note, a recent paper reported a targeted deletion of the renal proximal-tubule insulin-receptor in mice promoted hyperglycemia, up regulation of glucose 6 phosphatase and gluconeogenesis [112]. Collectively, this pattern is consistent with the stimulatory effects of acidic pH [146] and HYP mice osteoblasts have decreased pH and increased gluconeogenesis [52], [53]. Indeed, the increased acidic ASARM-peptide levels in HYP mice kidney, bone, teeth, urine and circulation [7], [54], [55], [58], [59], [62], [64], [66], [70], [71], [140], [141], [147], [148] likely contributes to the lowered pH and thus plays a major pathophysiological role.

The marked suppression of GAPDH expression in HYP bone and kidney (this study) with a suppressed Pentose Phosphate Pathway (PPP) [50], [123] is intriguing. Specifically, inactivation of GAPDH by oxidants is reported to induce a temporal re-routing of metabolic flux from glycolysis to the PPP pathway [149], [150]. The reduction in HYP mice PPP activity would be expected to decrease the available reduced-NADPH for fatty acid synthesis and also for cytochrome P450 hydroxylases. The latter is consistent with the reduced HYP-VE fat mass and the former is consistent with suppressed HYP-VE mice NADPH-dependent 1 α-hydroxylase activity and thus 1.25(OH)_2_D_3_ synthesis. As discussed earlier, we and others show an increased 1 α hydroxylase mRNA expression but reduced enzyme activity occurs with HYP mice [76]–[79]. This is consistent with ASARM mediated poisoning of 1 α hydroxylase enzyme activity with PPP suppression of NADPH production (1 α hydroxylase enzyme cofactor). Detoxification of reactive oxygen intermediates (ROS) would also be affected by the reduced NADPH availability leading to alterations in fatty acid metabolism. Specifically, NADPH is needed by several antioxidant-systems including glutaredoxin and thioredoxin as well as being essential for the recycling of glutathione, fatty acid synthesis, steroid synthesis (vitamin D metabolic enzymes; hydroxylases). Also, NADPH directly supplies electrons for the removal of superoxide via hydrogen peroxide and glutathione peroxidase.

The static and dynamic/temporal percentage change in fat-mass of HYP-VE mice as discussed earlier was significantly and markedly suppressed relative to WT-VE mice (Figures 6 and 7). In contrast, although no significant fat-mass differences in SPR4-treated WT-mice (WT-SPR4) relative to WT-VE mice occurred (static and dynamic), a significant increase in the fat-mass/weight ratio of WT-SPR4 mice did occur (static and dynamic). This contrasted with HYP-SPR4 mice relative to HYP-VE mice; these mice (HYP-SPR4) exhibited a marked and significant increase both in static and temporal fat-mass change over the 28 day treatment. These observations suggest a role for ASARM-peptides in mediating an abnormal and reduced fat deposition in HYP-mice. Of note, no significant differences in food uptake were observed between the WT-mice, HYP-mice and SPR4-treated mice of both groups (data not shown). Consistent with the reduction in HYP-VE dynamic fat-mass, reduced levels of Perilipin-2 (PLIN2) bone expression occurred with HYP-VE mice and WT-SPR4 mice. Furthermore, the temporal increase in dynamic fat-mass that occurred with HYP-SPR4 treated mice relative to HYP-VE mice was also accompanied by increased PLIN2 gene bone-expression. PLIN2 null-mice are reportedly protected against diet-induced obesity, adipose inflammation and fatty liver disease [90]. Missense mutations in humans affects lipolysis and is associated with reduced triglyceride (TAG) concentrations [151]. Increased expression of PLIN2 promotes cellular lipid accumulation in humans and mice [152], [153]. Also, two pathways for production of glycerol phosphate occur in liver and one in adipose tissue. Glycerol, in the form of glycerol phosphate is the initial acceptor of fatty acids in triacylglycerol (TAG) synthesis. TAGs are the major energy reserve of the body and are stored within adipocytes as coalesced micelles. In adipose tissue, glycerol phosphate is synthesized from the glycolytic intermediate dihydroxyacetone phosphate via NADH reduction and the enzyme glycerol phosphate dehydrogenase. In the liver, the same biosynthetic pathway occurs plus an additional route that does not occur in adipose tissue. Specifically, this second pathway involves the direct phosphorylation of glycerol using both ATP and glycerol kinase. Of relevance, adipocytes can only take up glucose in the presence of insulin. Therefore, the observed reduced fat mass (and implied reduced TAG) in HYP mice is consistent with the hypoinsulinemia, suppressed GAPDH (key glycolysis enzyme) and reported reduced ATP. Furthermore, the low circulating levels of insulin in the HYP-VE mice would also pre-dispose to an increased active adipocyte hormone-sensitive lipase (HSL), the enzyme responsible for release of fatty acids from TAG. However, HYP-VE circulating epinephrine levels are also reduced and in combination with reduced ATP this would predispose to reduced cAMP-dependent protein kinase activation of HSL. Thus, in combination, the reduced fat mass in HYP-VE mice is consistent with the altered metabolic and hormonal biochemistry that induces a reduced glycerol synthesis and altered fatty acid release from TAG. The insulin, glucose, leptin and epinephrine levels are completely corrected with HYP-SPR4 treated mice in line with the increased fat mass and PLIN2 expression. Since WT SPR4 treatment (PHEX inhibition) duplicates the changes found with HYP-mice, the SPR4 mediated corrections in HYP-VE mice (PHEX defective) are likely due to SPR4 sequestration of excess ASARM-peptides.

ATP depletion due to dietary hypophosphatemia induces AMP accumulation that is eventually degraded to uric acid that in mice is degraded further to allantoin by uricase (primates including humans lack functional uricase) [150]. HYP-mice however are hypouricemic and hypophosphatemic. Of note, we measured a major increase in the fractional excretion of uric acid and this may have been responsible for the net reduction in circulating uric acid. Also, since hyperinsulinemia has been shown to be associated with impaired renal UA clearance [154], [155] the suppressed HYP insulin levels is consistent with the HYP hypouricemia we observed. Furthermore, leptin is reported to positively correlate with serum uric acid levels and is a prime regulator of uric acid concentrations [156]–[158]. Thus, the hypoleptinemia we and others measured in HYP mice is also consistent with the HYP-mice suppressed uric acid levels. Our data also shows a marked reduction in HYP-mice fat mass that is independent of food intake. Since fatty acid (triglycerides) production and obesity are associated with the de novo synthesis of purine and accelerated UA production [40], [159]–[161] the low fat mass HYP-phenotype is congruent with the observed hypouricemia. Also, uric acid levels are reported to have a positive correlation with bone health and thus the HYP-mice hypouricemia is concordant with the impaired bone phenotype [37]–[39]. The dynamic increase in fat mass and corrected insulin, leptin and glucose in HYP mice treated with SPR4-peptide with accompanying reduced fractional excretion of uric acid is consistent with a role for ASARM-peptides. Of note, the fact that SPR4-peptide induces a marked suppression in uric acid levels in WT-VE mice is also of clinical interest given the well documented positive association for increased risk of heart disease, hypertension, kidney disease, metabolic syndrome, diabetes and adverse outcomes in these patients [40]. This effect is likely mediated through SPR4-peptide inhibition of WT PHEX activity.

Our study provides compelling support for a PHEX-DMP1-Integrin pathway that regulates FGF23 production and bone-energy mineral metabolism. The differential effect on select target genes between HYP-vehicle mice (HYP-VE) and Wild-type or HYP-Mice mice treated with SPR4-peptide (WT-SPR4 and HYP-SPR4) provided a unique opportunity to illustrate the bimodal nature of SPR4-peptide activity. Specifically SPR4-peptide is proposed to: (1) competitively inhibit PHEX activity in WT mice by binding to DMP1-ASARM motif and thus mimic key phenotypic changes seen in HYP-mice, (2) SPR4 enhances PHEX activity in WT-mice by binding to an endogenous inhibitor of PHEX, ASARM-peptide, (3) SPR4 suppresses the pathologic effects of increased ASARM-peptide levels that occur in HYP-mice by binding to free ASARM-peptide. Identical changes in serum uric-acid, sympathetic tone, adiponectin, leptin, serum phosphate, FGF23 and PTH occur with both HYP vehicle mice (HYP-VE) and WT mice infused with SPR4-peptide (WT-SPR4). This suggests a direct or indirect inhibition of PHEX by SPR4 is responsible for these alterations. Treatment of HYP-mice with SPR4 peptide resulted in striking beneficial changes in serum glucose, insulin, leptin, osteocalcin, sclerostin, fat mass, 1.25(OH)_2_D_3_, and calcium with a partial correction of the hypophosphatemia. Also, HYP-SPR4 mice showed a correction in uric acid renal handling, adiponectin and PTH. This was accompanied by markedly suppressed ENPP1 and FAM20C expression (both increased in HYP-VE mice). Since HYP-mice do not have functional PHEX, SPR4 mediated sequestration and neutralization of ASARM-peptide is likely responsible for these changes. Some of these positive changes also occurred with SPR4-peptide treated WT-mice (markedly suppressed sclerostin, FAM20C, ENPP1 and ESP for example). This supports a role for ASARM-peptides in normal physiology and points to a possible therapeutic utility for SPR4-peptide in familial rickets, diabetes, osteoporosis and obesity.

## Methods

### Animals, Procedures, Diets and Peptides

#### Ethics Statement

Mice (C57BL/6) were housed at the University of Kansas Medical Center under the supervision of the Department of Laboratory Animal Resources. The policies and procedures of the animal laboratory are in accordance with those detailed in the Guide for the Care and Use of Laboratory Animals published by the US Department of Health and Human Services (DHHS Publ. NIH 86–23, 1985). Procedural protocols were approved by the University of Kansas Institutional Animal Care and Use Committee.

#### Procedures, Diets and Peptides

Male (5-week) C57B/L6 mice that were wild-type (WT) or mutant X-linked Hypophosphatemic Rickets mice (HYP) were used for the study. All mice were maintained on a 1% phosphorus and 2.4 IU/g Vitamin-D3 diet (Harlan Teklad Rodent Diet 8604, Indianapolis, IN). Mice (5 week) were surgically implanted with Alzet osmotic pumps (Durect Corporation, Cupertino, CA) as described previously [54] and infused with SPR4-peptide (276 nmoles/hr/kg) or vehicle (0.9% physiological Saline; VE) for 28 days. Specifically, Alzet pump model #2004 with a constant infusion rate of 0.25 υL/h over 28 days was used. Four groups were studied (n = 6/group); (1) Wild type mice infused with vehicle (WT-VE), (2) HYP-mice infused with vehicle (HYP-VE), (3) Wild type mice infused with SPR4 peptide (WT-SPR4) and (4) HYP-mice infused with SPR4 peptide (HYP-SPR4). The SPR4 peptide (4.2 kDa) (NH2-TVNAFYSASTNYPRSLSYGAIGVIVGHEFTHGFDNNGRGENIADNG-OH) was synthesized using standard techniques by Polypeptide Laboratories (San Diego, CA 92126) as described previously [56], [66]. Peptide purity was greater than 80% via HPLC, ion-exchange and also mass spectrometry. SPR4-peptide was dissolved as follows; 100 υL/1 mg of peptide of 25 mM acetic acid was first added to dissolve the peptide, then 900 υL of 50 mM Tris pH 7.4/150 mM NaCl was added and after thorough mixing 20 υL of 1 mM ZnCl_2_. Note, it is important to add the ZnCl_2_ last to maintain peptide solubility. The final buffer composition was 44 mM Tris pH 7.4/132 mM NaCL/19.6 µM ZnCl_2_.

### Serum and Urine Analysis

At specific intervals throughout the experiment as detailed in the results section tail blood-samples were collected in serum-separator tubes and serum prepared as described previously [54]. On the final day of the infusion experiment (day 28) blood and urine was collected from mice fasted overnight in metabolic cages with full access to water (1 cage/mouse). The blood from the final bleed was collected by cardiac exsanguination and serum urinalysis carried out as described previously [54], [55], [64], [140], [162]. Briefly, Osteocalcin (Mouse Osteocalcin EIA Kit; BTI, Stoughton, MA), alkaline phosphatase (Liquid Alkaline Phosphatase; Pointe Scientific Inc, Canton, MI), 1.25(OH)_2_D_3_ (IDS Inc., Fountain Hills, AZ) and FGF23 (Kainos Laboratories Inc., Tokyo, Japan) were measured on serum samples. Inorganic phosphorus, calcium, creatinine (Pointe Scientific Inc, Canton, MI) and Osteopontin (Quantikine Mouse Osteopontin; R&D Systems, Minneapolis, MN) levels were assessed both in serum and urine. A competitive ELISA kit was used for the ASARM peptide measurement as previously published for serum and urine samples [7], [54], [140], [141].

### Bone and Fat mass Analysis: Dual X-Ray Absorptiometry (DEXA)

Dual X-Ray Absorptiometry (DEXA) using a PIXImus system (LUNAR Corporation, Madison, WI) was carried out as described previously [140]. A dual energy X-ray PIXImus densitometer (LUNAR Corporation, Madison, WI) was used for measuring fat mass, bone mineral density (BMD) and bone mineral content. Four different sites were determined by adjusting the region of interest (ROI): entire humerus, entire femur, entire tibia and L1 to L5 vertebrae. ROI was adjusted on each bone length and width. In addition, abdominal fat mass was evaluated in a ROI delineated by L1 and L5 vertebrae and including the whole body width a described previously [140]. Measurements were taken at baseline prior to treatment and at 4 weeks prior to sacrifice.

### RNA Isolation, tissue extraction, Real Time PCR, Immunohistochemistry (IHC) & Western Blotting

The above methods were performed as described previously for femurs and kidneys [54], [66], [140] with specific polyclonal primary antibodies and primers shown in Tables 6 and 7 respectively. Transferrin was used as an internal reference for all protocols (RNA and protein) since GAPDH was markedly suppressed in HYP-mice bone (-×6.2) and kidney (-×2.0). The Pfaffl mathematical model for the relative quantification of real-time PCR data was used to measure relative gene expression [163]. Immunohistochemistry on renal sections was carried out as described previously [7], [66], [140].

**Table 6:** Table of primary antibodies used in the study:

Primers for gene expression by RT-qPCR	
Antibody	Reference/Company
rabbit anti-MEPE (ASARM)	Bresler et al 2004 (8)
rabbit anti-MEPE (RGD)	Bresler et al 2004 (8)
goat anti-NPT2a	Santa Cruz Biotechnology, CA
rabbit anti-Transferrin	Abcam International Canada
goat anti-GAPDH	Santa Cruz Biotechnology, CA

**Table 7:** Table of primers used for quantitative RT-PCR (qRT-PCR).

	Primers for gene expression by RT-qPCR	
Gene	Forward	Reverse
Esp	5'-GACTCTCAGAAGATTCACAGTTGC-3'	5'-AAAGCCCAGGCTCAGGTT-3'
Phex	5'-TGCCAGAGAACAAGTGCAAA-3'	5'-CTAATGGCACCATTGACCCTA-3'
Vegf	5'-AAAAACGAAAGCGCAAGAAA-3'	5'-TTTCTCCGCTCTGAACAAGG-3'
Dmp1	5'-GGTTTTGACCTTGTGGGAAA-3'	5'-TTGGGATGCGATTCCTCTAC-3'
FGF23	5'-ATCTCCACGGCAACATTTTT-3'	5'-GTCCACTGGCGGAACTTG-3'
Plin2	5'-CTCCACTCCACTGTCCACCT-3'	5'-GCTTATCCTGAGCACCCTGA-3'
Bglap (osteocalcin)	5'-AGACTCCGGCGCTACCTT-3'	5'-CTCGTCACAAGCAGGGTTAAG-3'
Gapdh	5'-TGTCAAGCTCATTTCCTGGTATGA-3'	5'-CTTACTCCTTGGAGGCCATGTAG-3'
Sost (Sclerostin)	5'-TCCTGAGAACAACCAGACCA-3'	5'-GCAGCTGTACTCGGACACATC-3'
Mepe	5'-GATGCAGGCTGTGTCTGTTG-3'	5'-TGTCTTCATTCGGCATTGG-3
Cyclophilin	5'-CAGACGCCACTGTCGCTTT-3'	5'-TGTCTTTGGAACTTTGTCTGCAA-3'
24-hydroxylase	5'-AACTGTACGCTGCTGTCACG-3'	5'-AATCCACATCAAGCTGTTTGC-3'
1 α-hydroxylase	5'-AGTGGGGAATGTGACAGAGC-3'	5'-GGAGAGCGTATTGGATACCG-3'
NPT2c	5'-CAGCCCTGCAGACATGTTAAT-3	5'-GCACCAGGTACCACAGCAG-3'
NPT2a	5-GATTTGGTGTCACCCAGACA-3'	5'-ATGGCCTCTACCCTGGACAT-3'
Transferrin	5'-GACTCCGAACAACCTGAAGC-3'	5'-GCGTAGTAGTAGGTCTGTGGATGTT-3'
FAM20C	5'-TGAACAGCGACATCAGGTTT-3'	5'-CGCCTTCAGCACCTTCAT-3'
ENPP1	5'-CGGACGCTATGATTCCTTAGA-3'	5'-AGCACAATGAAGAAGTGAGTCG-3'

### Statistical analyses

Statistical analysis was performed using statistical software STATISTICA (StatSoft Inc., Tulsa, OK, USA) or PRISM5 (GraphPad Software inc., La Jolla, CA USA). Differences between groups were initially analyzed by two-way ANOVA. When F values for a given variable were found to be significant, the sequentially rejecting Bonferroni-Holm test was subsequently performed using the Holm's adjusted p values. For qRT-PCR gene analysis fold differences in expression calculated by the Pfaffl method [163] were statistically analyzed for significance using the One Sample t-test and the Wilcoxon Signed rank-test with theoretical means set to 1. Results for all tests were considered to be significantly different at p<0.05.

## References

[1] Yamaguchi T, Sugimoto T (2011) Bone metabolism and fracture risk in type 2 diabetes mellitus [Review]. Endocrine journal 58: 613–624. 21778617 10.1507/endocrj.ej11-0063

[2] Zhang L, Choi HJ, Estrada K, Leo PJ, Li J, et al. (2013) Multi-stage genome-wide association meta-analyses identified two new loci for bone mineral density. Hum Mol Genet [Epub ahead of print] PMID:24249740. 10.1093/hmg/ddt575 PMC3943521 24249740

[3] Hsu YH, Kiel DP (2012) Clinical review: Genome-wide association studies of skeletal phenotypes: what we have learned and where we are headed. J Clin Endocrinol Metab 97: E1958–1977. 22965941 10.1210/jc.2012-1890 PMC3674343

[4] Zmuda JM, Yerges-Armstrong LM, Moffett SP, Klei L, Kammerer CM, et al (2011) Genetic analysis of vertebral trabecular bone density and cross-sectional area in older men. Osteoporosis international 22: 1079–1090. 21153022 10.1007/s00198-010-1296-0 PMC3691107

[5] Jemtland R, Holden M, Reppe S, Olstad OK, Reinholt FP, et al (2011) Molecular disease map of bone characterizing the postmenopausal osteoporosis phenotype. Journal of bone and mineral research 26: 1793–1801. 21452281 10.1002/jbmr.396

[6] Styrkarsdottir U, Halldorsson BV, Gudbjartsson DF, Tang NL, Koh JM, et al (2010) European bone mineral density loci are also associated with BMD in East-Asian populations. PLoS One 5: e13217. 20949110 10.1371/journal.pone.0013217 PMC2951352

[7] Bresler D, Bruder J, Mohnike KL, Fraser D, Rowe PSN (2004) Serum MEPE-ASARM-peptides are elevated in X-linked rickets (HYP): implications for phosphaturia and rickets. J Endocrinol 183: R1–9. 15590969 10.1677/joe.1.05989 PMC3357083

[8] Jain A, Fedarko NS, Collins MT, Gelman R, Ankrom MA, et al (2004) Serum levels of matrix extracellular phosphoglycoprotein (MEPE) in normal humans correlate with serum phosphorus, parathyroid hormone and bone mineral density. J Clin Endocrinol Metab 89: 4158–4161. 15292364 10.1210/jc.2003-032031

[9] Confavreux CB, Levine RL, Karsenty G (2009) A paradigm of integrative physiology, the crosstalk between bone and energy metabolisms. Mol Cell Endocrinol 310: 21–29. 19376193 10.1016/j.mce.2009.04.004 PMC3667507

[10] Yadav VK, Oury F, Tanaka K, Thomas T, Wang Y, et al (2011) Leptin-dependent serotonin control of appetite: temporal specificity, transcriptional regulation, and therapeutic implications. J Exp Med 208: 41–52. 21187319 10.1084/jem.20101940 PMC3023132

[11] Karsenty G, Yadav VK (2011) Regulation of bone mass by serotonin: molecular biology and therapeutic implications. Annu Rev Med 62: 323–331. 21073335 10.1146/annurev-med-090710-133426

[12] Yadav VK, Oury F, Suda N, Liu ZW, Gao XB, et al (2009) A serotonin-dependent mechanism explains the leptin regulation of bone mass, appetite, and energy expenditure. Cell 138: 976–989. 19737523 10.1016/j.cell.2009.06.051 PMC2768582

[13] Yadav VK, Ryu JH, Suda N, Tanaka KF, Gingrich JA, et al (2008) Lrp5 controls bone formation by inhibiting serotonin synthesis in the duodenum. Cell 135: 825–837. 19041748 10.1016/j.cell.2008.09.059 PMC2614332

[14] Chabbi-Achengli Y, Coudert AE, Callebert J, Geoffroy V, Cote F, et al (2012) Decreased osteoclastogenesis in serotonin-deficient mice. Proc Natl Acad Sci U S A 109: 2567–2572. 22308416 10.1073/pnas.1117792109 PMC3289318

[15] Karsenty G (2001) Leptin controls bone formation through a hypothalamic relay. Recent Prog Horm Res 56: 401–415. 11237223 10.1210/rp.56.1.401

[16] Ducy P, Amling M, Takeda S, Priemel M, Schilling AF, et al (2000) Leptin inhibits bone formation through a hypothalamic relay: a central control of bone mass. Cell 100: 197–207. 10660043 10.1016/s0092-8674(00)81558-5

[17] Amling M, Takeda S, Karsenty G (2000) A neuro (endo)crine regulation of bone remodeling. Bioessays 22: 970–975. 11056473 10.1002/1521-1878(200011)22:11<970::AID-BIES3>3.0.CO;2-L

[18] Thrailkill KM, Jo CH, Cockrell GE, Moreau CS, Lumpkin CK Jr, et al (2012) Determinants of undercarboxylated and carboxylated osteocalcin concentrations in type 1 diabetes. Osteoporos Int 23: 1799–1806. 22068385 10.1007/s00198-011-1807-7 PMC3471372

[19] Sarkar PD, Choudhury AB (2012) Relationship of serum osteocalcin levels with blood glucose, insulin resistance and lipid profile in central Indian men with type 2 diabetes. Arch Physiol Biochem 118: 260–264. 22978420 10.3109/13813455.2012.715651

[20] Chen L, Li Q, Yang Z, Ye Z, Huang Y, et al (2012) Osteocalcin, glucose metabolism, lipid profile and chronic low-grade inflammation in middle-aged and elderly Chinese. Diabet Med 30: 309–317. 10.1111/j.1464-5491.2012.03769.x 22913521

[21] Chang MK, Kramer I, Keller H, Gooi JH, Collett C, et al (2013) Reversing LRP5-dependent osteoporosis and SOST-deficiency induced sclerosing bone disorders by altering WNT signaling activity. J Bone Miner Res 29: 29–42. 10.1002/jbmr.2059 23901037

[22] Cui Y, Niziolek PJ, Macdonald BT, Zylstra CR, Alenina N, et al (2011) Lrp5 functions in bone to regulate bone mass. Nature medicine 17: 684–691. 10.1038/nm.2388 PMC3113461 21602802

[23] Lee GS, Simpson C, Sun BH, Yao C, Foer D, et al (2013) Measurement of plasma, serum, and platelet serotonin in individuals with high bone mass and mutations in LRP5. J Bone Miner Res 29: 976–981. 10.1002/jbmr.2086 PMC3935983 24038240

[24] Boudin E, Jennes K, de Freitas F, Tegay D, Mortier G, et al (2013) No mutations in the serotonin related TPH1 and HTR1B genes in patients with monogenic sclerosing bone disorders. Bone 55: 52–56. 23563356 10.1016/j.bone.2013.03.015

[25] Lam DD, Leinninger GM, Louis GW, Garfield AS, Marston OJ, et al (2011) Leptin does not directly affect CNS serotonin neurons to influence appetite. Cell Metab 13: 584–591. 21531340 10.1016/j.cmet.2011.03.016 PMC3087147

[26] Hwang YC, Jee JH, Jeong IK, Ahn KJ, Chung HY, et al (2012) Circulating osteocalcin level is not associated with incident type 2 diabetes in middle-aged male subjects: mean 8.4-year retrospective follow-up study. Diabetes Care 35: 1919–1924. 22773701 10.2337/dc11-2471 PMC3424992

[27] Mori K, Emoto M, Motoyama K, Lee E, Yamada S, et al (2012) Undercarboxylated osteocalcin does not correlate with insulin resistance as assessed by euglycemic hyperinsulinemic clamp technique in patients with type 2 diabetes mellitus. Diabetol Metab Syndr 4: 53. 23249601 10.1186/1758-5996-4-53 PMC3565869

[28] Price S (2011) Bone: Evidence for local effects of LRP5 on bone mass. Nature reviews Rheumatology 7: 373. 10.1038/nrrheum.2011.84 21691327

[29] Goltzman D (2011) LRP5, serotonin and bone: Complexity, contradictions and conundrums. J Bone Miner and Res 26: 2002–2011. 21713997 10.1002/jbmr.462

[30] Idelevich A, Sato K, Baron R (2013) What are the effects of leptin on bone and where are they exerted? J Bone Miner Res 28: 18–21. 23188700 10.1002/jbmr.1812

[31] Turner RT, Kalra SP, Wong CP, Philbrick KA, Lindenmaier LB, et al (2013) Peripheral leptin regulates bone formation. J Bone Miner Res 28: 22–34. 22887758 10.1002/jbmr.1734 PMC3527690

[32] Hamrick MW, Della-Fera MA, Choi YH, Pennington C, Hartzell D, et al (2005) Leptin treatment induces loss of bone marrow adipocytes and increases bone formation in leptin-deficient ob/ob mice. J Bone Miner Res 20: 994–1001. 15883640 10.1359/JBMR.050103

[33] Steppan CM, Crawford DT, Chidsey-Frink KL, Ke H, Swick AG (2000) Leptin is a potent stimulator of bone growth in ob/ob mice. Regul Pept 92: 73–78. 11024568 10.1016/s0167-0115(00)00152-x

[34] Schwartz AV, Schafer AL, Grey A, Vittinghoff E, Palermo L, et al (2013) Effects of antiresorptive therapies on glucose metabolism: Results from the FIT, HORIZON-PFT, and FREEDOM trials. J Bone Miner Res 28: 1348–1354. 23322676 10.1002/jbmr.1865

[35] Nanes MS (2013) Phosphate wasting and fibroblast growth factor-23. Curr Opin Endocrinol Diabetes Obes 20: 523–531. 24157604 10.1097/01.med.0000436189.80104.80

[36] Johnson RJ, Andrews P (2010) Fructose, Uricase, and the Back-to-Africa Hypothesis. Evolutionary Anthropology 19: 250–257.

[37] Makovey J, Macara M, Chen JS, Hayward CS, March L, et al (2013) Serum uric acid plays a protective role for bone loss in peri- and postmenopausal women: a longitudinal study. Bone 52: 400–406. 23111314 10.1016/j.bone.2012.10.025

[38] Sritara C, Ongphiphadhanakul B, Chailurkit L, Yamwong S, Ratanachaiwong W, et al (2012) Serum Uric Acid Levels in Relation to Bone-Related Phenotypes in Men and Women. J Clin Densitom 16: 336–340. 22727551 10.1016/j.jocd.2012.05.008

[39] Nabipour I, Sambrook PN, Blyth FM, Janu MR, Waite LM, et al (2011) Serum uric acid is associated with bone health in older men: a cross-sectional population-based study. J Bone Miner Res 26: 955–964. 21541998 10.1002/jbmr.286

[40] Kanbay M, Segal M, Afsar B, Kang DH, Rodriguez-Iturbe B, et al (2013) The role of uric acid in the pathogenesis of human cardiovascular disease. Heart 99: 759–766. 23343689 10.1136/heartjnl-2012-302535

[41] Feig DI, Kang DH, Johnson RJ (2008) Uric acid and cardiovascular risk. N Engl J Med 359: 1811–1821. 18946066 10.1056/NEJMra0800885 PMC2684330

[42] Sluijs I, Beulens JW, van der AD, Spijkerman AM, Schulze MB, et al (2013) Plasma uric acid is associated with increased risk of type 2 diabetes independent of diet and metabolic risk factors. J Nutr 143: 80–85. 23173177 10.3945/jn.112.167221

[43] Stellato D, Morrone LF, Di Giorgio C, Gesualdo L (2012) Uric acid: a starring role in the intricate scenario of metabolic syndrome with cardio-renal damage? Intern Emerg Med 7: 5–8. 21842242 10.1007/s11739-011-0642-3

[44] Santos RD (2012) Elevated uric acid, the metabolic syndrome and cardiovascular disease: cause, consequence, or just a not so innocent bystander? Endocrine 41: 350–352. 22451439 10.1007/s12020-012-9657-4

[45] Grassi D, Ferri L, Desideri G, Di Giosia P, Cheli P, et al (2012) Chronic Hyperuricemia, Uric Acid Deposit and Cardiovascular Risk. Curr Pharm Des 19: 2432–2438. 10.2174/1381612811319130011 PMC3606968 23173592

[46] Civantos Modino S, Guijarro de Armas MG, Monereo Mejias S, Montano Martinez JM, Iglesias Bolanos P, et al (2012) Hyperuricemia and metabolic syndrome in children with overweight and obesity. Endocrinol Nutr 59: 533–538. 23089370 10.1016/j.endonu.2012.06.010

[47] Robles-Cervantes JA, Ramos-Zavala MG, Gonzalez-Ortiz M, Martinez-Abundis E, Valencia-Sandoval C, et al (2011) Relationship between Serum Concentration of Uric Acid and Insulin Secretion among Adults with Type 2 Diabetes Mellitus. Int J Endocrinol 2011: 107904. 22216028 10.1155/2011/107904 PMC3246727

[48] HYP-consortium, Francis F, Hennig S, Korn b, Reinhardt R, et al (1995) A gene (PEX) with homologies to endopeptidases is mutated in patients with X-linked hypophosphatemic rickets. The HYP Consortium. Nat Genet 11: 130–136. 7550339 10.1038/ng1095-130

[49] Vaughn LK, Meyer RA Jr, Meyer MH (1986) Increased metabolic rate in X-linked hypophosphatemic mice. Endocrinology 118: 441–445. 3940855 10.1210/endo-118-1-441

[50] Capparelli AW, Roh D, Dhiman JK, Jo OD, Yanagawa N (1992) Altered proximal tubule glucose metabolism in X-linked hypophosphatemic mice. Endocrinology 130: 328–334. 1309337 10.1210/endo.130.1.1309337

[51] Nesbitt T, Econs MJ, Byun JK, Martel J, Tenenhouse HS, et al (1995) Phosphate transport in immortalized cell cultures from the renal proximal tubule of normal and Hyp mice: evidence that the HYP gene locus product is an extrarenal factor. JBoneMinerRes 10: 1327–1333. 10.1002/jbmr.5650100909 7502704

[52] Rifas L, Gupta A, Hruska KA, Avioli LV (1995) Altered osteoblast gluconeogenesis in X-linked hypophosphatemic mice is associated with a depressed intracellular pH. CalcifTissue Int 57: 60–63. 10.1007/BF00298998 7671167

[53] Xie W, Mechin MC, Dubois SG, Lajeunesse D, van de Werve G (2002) Up-regulation of liver glucose-6-phosphatase in x-linked hypophosphatemic mice. Horm Metab Res 34: 288–292. 12173068 10.1055/s-2002-33256

[54] David V, Martin AC, Hedge AM, Drezner MK, Rowe PS (2011) ASARM peptides: PHEX-dependent & independent regulation of serum phosphate. Am J Physiol Renal Physiol 300: F783–791. 21177780 10.1152/ajprenal.00304.2010 PMC3064126

[55] Rowe PSN, Garrett IR, Schwarz PM, Carnes DL, Lafer EM, et al (2005) Surface Plasmon Resonance (SPR) confirms MEPE binds to PHEX via the MEPE-ASARM-motif: A model for impaired mineralization in X-linked rickets (HYP). Bone 36: 33–46. 15664000 10.1016/j.bone.2004.09.015 PMC3361744

[56] Atkins GJ, Rowe PS, Lim HP, Welldon KJ, Ormsby R, et al (2011) Sclerostin is a locally acting regulator of late-osteoblast/pre-osteocyte differentiation and regulates mineralization through a MEPE-ASARM dependent mechanism. J Bone Miner Res 26: 1425–1436. 21312267 10.1002/jbmr.345 PMC3358926

[57] Campos M, Couture C, Hirata IY, Juliano MA, Loisel TP, et al (2003) Human recombinant endopeptidase PHEX has a strict S1' specificity for acidic residues and cleaves peptides derived from fibroblast growth factor-23 and matrix extracellular phosphoglycoprotein. Biochem J 373: 271–279. 12678920 10.1042/BJ20030287 PMC1223479

[58] Salmon B, Bardet C, Khaddam M, Naji J, Coyac BR, et al (2013) MEPE-Derived ASARM Peptide Inhibits Odontogenic Differentiation of Dental Pulp Stem Cells and Impairs Mineralization in Tooth Models of X-Linked Hypophosphatemia. PLoS One 8: e56749. 23451077 10.1371/journal.pone.0056749 PMC3579870

[59] Barros NM, Hoac B, Neves RL, Addison WN, Assis DM, et al (2013) Proteolytic processing of osteopontin by PHEX and accumulation of osteopontin fragments in Hyp mouse bone, the murine model of X-linked hypophosphatemia. J Bone Miner Res 28: 688–699. 22991293 10.1002/jbmr.1766

[60] Addison W, Masica D, Gray J, McKee MD (2009) Phosphorylation-Dependent Inhibition of Mineralization by Osteopontin ASARM Peptides is Regulated by PHEX Cleavage. J Bone Miner Res 25: 695–705. 10.1359/jbmr.090832 19775205

[61] Addison W, Nakano Y, Loisel T, Crine P, McKee M (2008) MEPE-ASARM Peptides Control Extracellular Matrix Mineralization by Binding to Hydroxyapatite - An Inhibition Regulated by PHEX Cleavage of ASARM. J Bone Miner Res 23: 1638–1649. 18597632 10.1359/jbmr.080601

[62] Boukpessi T, Gaucher C, Leger T, Salmon B, Le Faouder J, et al (2010) Abnormal presence of the matrix extracellular phosphoglycoprotein-derived acidic serine- and aspartate-rich motif peptide in human hypophosphatemic dentin. Am J Pathol 177: 803–812. 20581062 10.2353/ajpath.2010.091231 PMC2913338

[63] Staines KA, Mackenzie NC, Clarkin CE, Zelenchuk L, Rowe PS, et al (2012) MEPE is a novel regulator of growth plate cartilage mineralization. Bone 51: 418–430. 22766095 10.1016/j.bone.2012.06.022 PMC3427007

[64] Rowe PS, Matsumoto N, Jo OD, Shih RN, Oconnor J, et al (2006) Correction of the mineralization defect in hyp mice treated with protease inhibitors CA074 and pepstatin. Bone 39: 773–786. 16762607 10.1016/j.bone.2006.04.012 PMC3358922

[65] Rowe PS, Liu S, Vierthaler L, Zhou J, Quarles LD (2007) Phosphorylated acidic serine-aspartate-rich MEPE-associated motif peptide from matrix extracellular phosphoglycoprotein inhibits phosphate regulating gene with homologies to endopeptidases on the X-chromosome enzyme activity. J Endocrinol 192: 261–267 (PR and SL Joint first authors). 17210763 10.1677/joe.1.07059 PMC3357085

[66] Martin A, David V, Laurence JS, Schwarz PM, Lafer EM, et al (2008) Degradation of MEPE, DMP1, and release of SIBLING ASARM-peptides (minhibins): ASARM-peptide(s) are directly responsible for defective mineralization in HYP. Endocrinology 149: 1757–1772. 18162525 10.1210/en.2007-1205 PMC2276704

[67] Rowe PS (2012) Regulation of Bone-Renal Mineral and Energy Metabolism: The PHEX, FGF23, DMP1, MEPE ASARM Pathway. Critical reviews in eukaryotic gene expression 22: 61–86. 22339660 10.1615/critreveukargeneexpr.v22.i1.50 PMC3362997

[68] Lorenz-Depiereux B, Bastepe M, Benet-Pages A, Amyere M, Wagenstaller J, et al (2006) DMP1 mutations in autosomal recessive hypophosphatemia implicate a bone matrix protein in the regulation of phosphate homeostasis. Nat Genet 38: 1248–1250. 17033625 10.1038/ng1868 PMC5942547

[69] Feng JQ, Ward LM, Liu S, Lu Y, Xie Y, et al (2006) Loss of DMP1 causes rickets and osteomalacia and identifies a role for osteocytes in mineral metabolism. Nat Genet 38: 1310–1315. 17033621 10.1038/ng1905 PMC1839871

[70] Boukpessi T, Septier D, Bagga S, Garabedian M, Goldberg M, et al (2006) Dentin alteration of deciduous teeth in human hypophosphatemic rickets. Calcif Tissue Int 79: 294–300. 17115324 10.1007/s00223-006-0182-4

[71] Boskey AL, Chiang P, Fermanis A, Brown J, Taleb H, et al (2010) MEPE's Diverse Effects on Mineralization. Calcif Tissue Int 86: 42–46. 19998030 10.1007/s00223-009-9313-z PMC2810528

[72] Rafaelsen SH, Raeder H, Fagerheim AK, Knappskog P, Carpenter TO, et al (2013) Exome sequencing reveals FAM20c mutations associated with fibroblast growth factor 23-related hypophosphatemia, dental anomalies, and ectopic calcification. J Bone Miner Res 28: 1378–1385. 23325605 10.1002/jbmr.1850

[73] Wang X, Wang S, Li C, Gao T, Liu Y, et al (2012) Inactivation of a novel FGF23 regulator, FAM20C, leads to hypophosphatemic rickets in mice. PLoS Genet 8: e1002708. 22615579 10.1371/journal.pgen.1002708 PMC3355082

[74] Vogel P, Hansen GM, Read RW, Vance RB, Thiel M, et al (2012) Amelogenesis Imperfecta and Other Biomineralization Defects in Fam20a and Fam20c Null Mice. Veterinary pathology 49: 998–1017. 22732358 10.1177/0300985812453177

[75] Tagliabracci VS, Engel JL, Wen J, Wiley SE, Worby CA, et al (2012) Secreted kinase phosphorylates extracellular proteins that regulate biomineralization. Science 336: 1150–1153. 22582013 10.1126/science.1217817 PMC3754843

[76] Ranch D, Zhang MY, Portale AA, Perwad F (2011) Fibroblast growth factor 23 regulates renal 1,25-dihydroxyvitamin D and phosphate metabolism via the MAP kinase signaling pathway in Hyp mice. Journal of bone and mineral research 26: 1883–1890. 21472778 10.1002/jbmr.401 PMC4409871

[77] Yuan B, Feng JQ, Bowman S, Liu Y, Blank RD, et al (2013) Hexa-D-Arginine treatment increases 7B2*PC2 activity in hyp-mouse osteoblasts and rescues the HYP phenotype. Journal of bone and mineral research 28: 56–72. 22886699 10.1002/jbmr.1738 PMC3523095

[78] Fujiwara I, Aravindan R, Horst RL, Drezner MK (2003) Abnormal regulation of renal 25-hydroxyvitamin D-1alpha-hydroxylase activity in X-linked hypophosphatemia: a translational or post-translational defect. J Bone Miner Res 18: 434–442. 12619927 10.1359/jbmr.2003.18.3.434

[79] Azam N, Zhang MY, Wang X, Tenenhouse HS, Portale AA (2003) Disordered regulation of renal 25-hydroxyvitamin D-1alpha-hydroxylase gene expression by phosphorus in X-linked hypophosphatemic (hyp) mice. Endocrinology 144: 3463–3468. 12865326 10.1210/en.2003-0255

[80] Eleftheriadis T, Antoniadi G, Pissas G, Liakopoulos V, Stefanidis I (2013) The renal endothelium in diabetic nephropathy. Ren Fail 35: 592–599. 23472883 10.3109/0886022X.2013.773836

[81] Tarr JM, Kaul K, Chopra M, Kohner EM, Chibber R (2013) Pathophysiology of Diabetic Retinopathy. ISRN Ophthalmol 2013: 343560. 24563789 10.1155/2013/343560 PMC3914226

[82] Millan JL (2012) The Role of Phosphatases in the Initiation of Skeletal Mineralization. Calcif Tissue Int 93: 299–306. 23183786 10.1007/s00223-012-9672-8 PMC3594124

[83] Saito T, Shimizu Y, Hori M, Taguchi M, Igarashi T, et al. (2011) A patient with hypophosphatemic rickets and ossification of posterior longitudinal ligament caused by a novel homozygous mutation in ENPP1 gene. Bone Epub PMID:21745613. 10.1016/j.bone.2011.06.029 21745613

[84] Lorenz-Depiereux B, Schnabel D, Tiosano D, Hausler G, Strom TM (2010) Loss-of-Function ENPP1 Mutations Cause Both Generalized Arterial Calcification of Infancy and Autosomal-Recessive Hypophosphatemic Rickets. Am J Hum Genet 86: 267–272. 20137773 10.1016/j.ajhg.2010.01.006 PMC2820166

[85] Lee NK, Sowa H, Hinoi E, Ferron M, Ahn JD, et al (2007) Endocrine regulation of energy metabolism by the skeleton. Cell 130: 456–469. 17693256 10.1016/j.cell.2007.05.047 PMC2013746

[86] Abate N, Chandalia M, Di Paola R, Foster DW, Grundy SM, et al (2006) Mechanisms of disease: Ectonucleotide pyrophosphatase phosphodiesterase 1 as a 'gatekeeper' of insulin receptors. Nat Clin Pract Endocrinol Metab 2: 694–701. 17143316 10.1038/ncpendmet0367

[87] Dong H, Maddux BA, Altomonte J, Meseck M, Accili D, et al (2005) Increased hepatic levels of the insulin receptor inhibitor, PC-1/NPP1, induce insulin resistance and glucose intolerance. Diabetes 54: 367–372. 15677494 10.2337/diabetes.54.2.367

[88] Prudente S, Morini E, Trischitta V (2009) Insulin signaling regulating genes: effect on T2DM and cardiovascular risk. Nat Rev Endocrinol 5: 682–693. 19924153 10.1038/nrendo.2009.215

[89] de Lorenzo C, Greco A, Fiorentino TV, Mannino GC, Hribal ML (2013) Variants of insulin-signaling inhibitor genes in type 2 diabetes and related metabolic abnormalities. Int J Genomics 2013: 376454. 23762820 10.1155/2013/376454 PMC3674720

[90] McManaman JL, Bales ES, Orlicky DJ, Jackman M, Maclean PS, et al (2013) Perilipin-2 Null Mice are Protected Against Diet-Induced Obesity, Adipose Inflammation and Fatty Liver Disease. J Lipid Res 54: 1346–1359. 23402988 10.1194/jlr.M035063 PMC3622329

[91] Billaudel B, Barakat L, Faure-Dussert A (1998) Vitamin D3 deficiency and alterations of glucose metabolism in rat endocrine pancreas. Diabetes Metab 24: 344–350. 9805645

[92] Hesse M, Frohlich LF, Zeitz U, Lanske B, Erben RG (2007) Ablation of vitamin D signaling rescues bone, mineral, and glucose homeostasis in Fgf-23 deficient mice. Matrix Biol 26: 20. 17123805 10.1016/j.matbio.2006.10.003

[93] Tiosano D, Schwartz Y, Braver Y, Hadash A, Gepstein V, et al (2011) The renin-angiotensin system, blood pressure, and heart structure in patients with hereditary vitamin D-resistance rickets (HVDRR). Journal of bone and mineral research 26: 2252–2260. 21590741 10.1002/jbmr.431

[94] Zeitz U, Weber K, Soegiarto DW, Wolf E, Balling R, et al (2003) Impaired insulin secretory capacity in mice lacking a functional vitamin D receptor. Faseb J 17: 509–511. 12551842 10.1096/fj.02-0424fje

[95] Haap M, Heller E, Thamer C, Tschritter O, Stefan N, et al (2006) Association of serum phosphate levels with glucose tolerance, insulin sensitivity and insulin secretion in non-diabetic subjects. Eur J Clin Nutr 60: 734–739. 16391583 10.1038/sj.ejcn.1602375

[96] Kalaitzidis R, Tsimihodimos V, Bairaktari E, Siamopoulos KC, Elisaf M (2005) Disturbances of phosphate metabolism: another feature of metabolic syndrome. Am J Kidney Dis 45: 851–858. 15861350 10.1053/j.ajkd.2005.01.005

[97] Xie W, Tran TL, Finegood DT, van de Werve G (2000) Dietary P(i) deprivation in rats affects liver cAMP, glycogen, key steps of gluconeogenesis and glucose production. Biochem J 352 Pt 1: 227–232. PMC1221451 11062077

[98] Paula FJ, Plens AE, Foss MC (1998) Effects of hypophosphatemia on glucose tolerance and insulin secretion. Horm Metab Res 30: 281–284. 9660090 10.1055/s-2007-978884

[99] Ditzel J, Lervang HH (2009) Disturbance of inorganic phosphate metabolism in diabetes mellitus: temporary therapeutic intervention trials. Diabetes Metab Syndr Obes 2: 173–177. 21437131 PMC3048005

[100] Cade C, Norman AW (1986) Vitamin D3 improves impaired glucose tolerance and insulin secretion in the vitamin D-deficient rat in vivo. Endocrinology 119: 84–90. 3013599 10.1210/endo-119-1-84

[101] Cade C, Norman AW (1987) Rapid normalization/stimulation by 1,25-dihydroxyvitamin D3 of insulin secretion and glucose tolerance in the vitamin D-deficient rat. Endocrinology 120: 1490–1497. 3549262 10.1210/endo-120-4-1490

[102] Hruska KA, Rifas L, Cheng SL, Gupta A, Halstead L, et al (1995) X-linked hypophosphatemic rickets and the murine Hyp homologue. AmJPhysiol 268: F357–F362. 10.1152/ajprenal.1995.268.3.F357 7900834

[103] DeFronzo RA, Lang R (1980) Hypophosphatemia and glucose intolerance: evidence for tissue insensitivity to insulin. N Engl J Med 303: 1259–1263. 6999353 10.1056/NEJM198011273032203

[104] Massry SG, Fadda GZ, Zhou XJ, Chandrasoma P, Cheng L, et al (1991) Impaired insulin secretion of aging: role of renal failure and hyperparathyroidism. Kidney Int 40: 662–667. 1745015 10.1038/ki.1991.258

[105] Joshi A, Varthakavi P, Chadha M, Bhagwat N (2013) A study of bone mineral density and its determinants in type 1 diabetes mellitus. J Osteoporos 2013: 397814. 23607045 10.1155/2013/397814 PMC3628496

[106] Lee B, Shao J (2013) Adiponectin and energy homeostasis. Rev Endocr Metab Disord. 10.1007/s11154-013-9283-3 PMC4006341 24170312

[107] Staines KA, Zhu D, Farquharson C, Macrae VE (2013) Identification of novel regulators of osteoblast matrix mineralization by time series transcriptional profiling. J Bone Miner Metab [Epub ahead of print]: PMID:23925391. 10.1007/s00774-013-0493-2 23925391

[108] Kanazawa I, Yamaguchi T, Yano S, Yamauchi M, Yamamoto M, et al (2007) Adiponectin and AMP kinase activator stimulate proliferation, differentiation, and mineralization of osteoblastic MC3T3-E1 cells. BMC Cell Biol 8: 51. 18047638 10.1186/1471-2121-8-51 PMC2214728

[109] Zhou XJ, Fadda GZ, Perna AF, Massry SG (1991) Phosphate depletion impairs insulin secretion by pancreatic islets. Kidney Int 39: 120–128. 1848327 10.1038/ki.1991.15

[110] Xie W, Li Y, Mechin MC, Van De Werve G (1999) Up-regulation of liver glucose-6-phosphatase in rats fed with a P(i)-deficient diet. Biochem J 343 Pt 2: 393–396. 10510305 10.1042/bj3430393 PMC1220566

[111] Trinh KY, O'Doherty RM, Anderson P, Lange AJ, Newgard CB (1998) Perturbation of fuel homeostasis caused by overexpression of the glucose-6-phosphatase catalytic subunit in liver of normal rats. J Biol Chem 273: 31615–31620. 9813078 10.1074/jbc.273.47.31615

[112] Tiwari S, Singh RS, Li L, Tsukerman S, Godbole M, et al (2013) Deletion of the insulin receptor in the proximal tubule promotes hyperglycemia. J Am Soc Nephrol 24: 1209–1214. 23723425 10.1681/ASN.2012060628 PMC3736710

[113] Haglin L (2001) Hypophosphataemia: cause of the disturbed metabolism in the metabolic syndrome. Med Hypotheses 56: 657–663. 11399116 10.1054/mehy.2000.1272

[114] Allon M (1992) Effects of insulin and glucose on renal phosphate reabsorption: interactions with dietary phosphate. J Am Soc Nephrol 2: 1593–1600. 1610980 10.1681/ASN.V2111593

[115] Hale LJ, Coward RJ (2013) Insulin signalling to the kidney in health and disease. Clin Sci (Lond) 124: 351–370. 23190266 10.1042/CS20120378

[116] Marsenic O (2009) Glucose control by the kidney: an emerging target in diabetes. Am J Kidney Dis 53: 875–883. 19324482 10.1053/j.ajkd.2008.12.031

[117] Gerich JE, Meyer C, Woerle HJ, Stumvoll M (2001) Renal gluconeogenesis: its importance in human glucose homeostasis. Diabetes Care 24: 382–391. 11213896 10.2337/diacare.24.2.382

[118] Wojcik M, Dolezal-Oltarzewska K, Janus D, Drozdz D, Sztefko K, et al (2012) FGF23 contributes to insulin sensitivity in obese adolescents - preliminary results. Clin Endocrinol (Oxf) 77: 537–540. 22103239 10.1111/j.1365-2265.2011.04299.x

[119] Wojcik M, Janus D, Dolezal-Oltarzewska K, Drozdz D, Sztefko K, et al (2012) The association of FGF23 levels in obese adolescents with insulin sensitivity. J Pediatr Endocrinol Metab 25: 687–690. 23155694 10.1515/jpem-2012-0064

[120] Levi E, Fadda GZ, Ozbasli C, Massry SG (1992) Evolution of metabolic and functional derangements of pancreatic islets in phosphate depletion. Endocrinology 131: 2182–2188. 1330495 10.1210/endo.131.5.1330495

[121] Travis SF, Sugerman HJ, Ruberg RL, Dudrick SJ, Delivoria-Papadopoulos M, et al (1971) Alterations of red-cell glycolytic intermediates and oxygen transport as a consequence of hypophosphatemia in patients receiving intravenous hyperalimentation. N Engl J Med 285: 763–768. 4998555 10.1056/NEJM197109302851402

[122] Hettleman BD, Sabina RL, Drezner MK, Holmes EW, Swain JL (1983) Defective adenosine triphosphate synthesis. An explanation for skeletal muscle dysfunction in phosphate-deficient mice. J Clin Invest 72: 582–589. 6874957 10.1172/JCI111006 PMC1129216

[123] Sabina RL, Drezner MK, Holmes EW (1982) Reduced renal cortical ribonucleoside triphosphate pools in three different hypophosphatemic animal models. Biochem Biophys Res Commun 109: 649–655. 7159438 10.1016/0006-291x(82)91989-1

[124] Ferron M, Wei J, Yoshizawa T, Del Fattore A, DePinho RA, et al (2010) Insulin signaling in osteoblasts integrates bone remodeling and energy metabolism. Cell 142: 296–308. 20655470 10.1016/j.cell.2010.06.003 PMC2910411

[125] Wu YY, Yu T, Zhang XH, Liu YS, Li F, et al (2012) 1,25(OH)2D3 inhibits the deleterious effects induced by high glucose on osteoblasts through undercarboxylated osteocalcin and insulin signaling. J Steroid Biochem Mol Biol 132: 112–119. 22595150 10.1016/j.jsbmb.2012.05.002

[126] Kode A, Mosialou I, Silva BC, Joshi S, Ferron M, et al (2012) FoxO1 protein cooperates with ATF4 protein in osteoblasts to control glucose homeostasis. J Biol Chem 287: 8757–8768. 22298775 10.1074/jbc.M111.282897 PMC3308768

[127] Li Q, Guo H, Chou DW, Berndt A, Sundberg JP, et al (2013) Mutant Enpp1asj mice as a model for generalized arterial calcification of infancy. Dis Model Mech 6: 1227–1235. 23798568 10.1242/dmm.012765 PMC3759342

[128] Mackenzie NC, Zhu D, Milne EM, van 't Hof R, Martin A, et al (2012) Altered bone development and an increase in FGF-23 expression in Enpp1(−/−) mice. PLoS One 7: e32177. 22359666 10.1371/journal.pone.0032177 PMC3281127

[129] Huitema LF, Apschner A, Logister I, Spoorendonk KM, Bussmann J, et al (2012) Entpd5 is essential for skeletal mineralization and regulates phosphate homeostasis in zebrafish. Proc Natl Acad Sci U S A 109: 21372–21377. 23236130 10.1073/pnas.1214231110 PMC3535636

[130] Sortica DA, Crispim D, Zaffari GP, Friedman R, Canani LH (2011) The role of ecto-nucleotide pyrophosphatase/phosphodiesterase 1 in diabetic nephropathy. Arq Bras Endocrinol Metabol 55: 677–685. 22231969 10.1590/s0004-27302011000900002

[131] Pan W, Ciociola E, Saraf M, Tumurbaatar B, Tuvdendorj D, et al (2011) Metabolic consequences of ENPP1 overexpression in adipose tissue. Am J Physiol Endocrinol Metab 301: E901–911. 21810932 10.1152/ajpendo.00087.2011 PMC3275110

[132] Jankowski M, Piwkowska A, Rogacka D, Audzeyenka I, Janaszak-Jasiecka A, et al (2011) Expression of membrane-bound NPP-type ecto-phosphodiesterases in rat podocytes cultured at normal and high glucose concentrations. Biochem Biophys Res Commun 416: 64–69. 22086174 10.1016/j.bbrc.2011.10.144

[133] Maddux BA, Chang YN, Accili D, McGuinness OP, Youngren JF, et al (2006) Overexpression of the insulin receptor inhibitor PC-1/ENPP1 induces insulin resistance and hyperglycemia. Am J Physiol Endocrinol Metab 290: E746–749. 16278247 10.1152/ajpendo.00298.2005

[134] Goldfine ID, Maddux BA, Youngren JF, Reaven G, Accili D, et al (2008) The role of membrane glycoprotein plasma cell antigen 1/ectonucleotide pyrophosphatase phosphodiesterase 1 in the pathogenesis of insulin resistance and related abnormalities. Endocr Rev 29: 62–75. 18199690 10.1210/er.2007-0004 PMC2244935

[135] Ishikawa HO, Xu A, Ogura E, Manning G, Irvine KD (2012) The Raine syndrome protein FAM20C is a Golgi kinase that phosphorylates bio-mineralization proteins. PLoS One 7: e42988. 22900076 10.1371/journal.pone.0042988 PMC3416761

[136] Tagliabracci A, Engel JL, Wiley SE, Xiao J, Gonzalez DJ, et al. (2014) Dynamic regulation of FGF23 by Fam20C phosphorylation, GalNAc-T3 glycosylation, and furin proteolysis. PNAS EPub Online. 10.1073/pnas.1402218111 PMC3992636 24706917

[137] Yancovitch A, Hershkovitz D, Indelman M, Galloway P, Whiteford M, et al (2011) Novel mutations in GALNT3 causing hyperphosphatemic familial tumoral calcinosis. Journal of bone and mineral metabolism 29: 621–625. 21347749 10.1007/s00774-011-0260-1

[138] Hirao M, Hashimoto J, Yamasaki N, Ando W, Tsuboi H, et al (2007) Oxygen tension is an important mediator of the transformation of osteoblasts to osteocytes. J Bone Miner Metab 25: 266–276. 17704991 10.1007/s00774-007-0765-9

[139] Bonewald LF (2002) Osteocytes: a proposed multifunctional bone cell. J Musculoskelet Neuronal Interact 2: 239–241. 15758443

[140] David V, Martin A, Hedge AM, Rowe PS (2009) Matrix extracellular phosphoglycoprotein (MEPE) is a new bone renal hormone and vascularization modulator. Endocrinology 150: 4012–4023. 19520780 10.1210/en.2009-0216 PMC2819738

[141] Yuan B, Takaiwa M, Clemens TL, Feng JQ, Kumar R, et al (2008) Aberrant Phex function in osteoblasts and osteocytes alone underlies murine X-linked hypophosphatemia. J Clin Invest 118: 722–734. 18172553 10.1172/JCI32702 PMC2157563

[142] Alexander MC, Lomanto M, Nasrin N, Ramaika C (1988) Insulin stimulates glyceraldehyde-3-phosphate dehydrogenase gene expression through cis-acting DNA sequences. Proc Natl Acad Sci U S A 85: 5092–5096. 2839830 10.1073/pnas.85.14.5092 PMC281694

[143] Alexander-Bridges M, Dugast I, Ercolani L, Kong XF, Giere L, et al (1992) Multiple insulin-responsive elements regulate transcription of the GAPDH gene. Adv Enzyme Regul 32: 149–159. 1386708 10.1016/0065-2571(92)90014-q

[144] Nesbitt T, Byun JK, Drezner MK (1996) Normal phosphate transport in cells from the S2 and S3 segments of Hyp-mouse proximal renal tubules. Endocrinology 137: 943–948. 8603607 10.1210/endo.137.3.8603607

[145] Roobol A, Alleyne GA (1973) Regulation of renal gluconeogenesis by calcium ions, hormones and adenosine 3':5'-cyclic monophosphate. Biochem J 134: 157–165. 4353084 10.1042/bj1340157 PMC1177796

[146] Kurokawa K, Rasmussen H (1973) Ioni control of renal gluconeogenesis. I. The interrelated effect of calcium and hydrogen ions. Biochim Biophys Acta 313: 17–31. 4745675 10.1016/0304-4165(73)90185-2

[147] Gaucher C, Boukpessi T, Septier D, Jehan F, Rowe PS, et al (2009) Dentin noncollagenous matrix proteins in familial hypophosphatemic rickets. Cells Tissues Organs 189: 219–223. 18701809 10.1159/000151382 PMC3352030

[148] Opsahl Vital S, Gaucher C, Bardet C, Rowe PS, George A, et al (2012) Tooth dentin defects reflect genetic disorders affecting bone mineralization. Bone 50: 989–997. 22296718 10.1016/j.bone.2012.01.010 PMC3345892

[149] Ralser M, Wamelink MM, Kowald A, Gerisch B, Heeren G, et al (2007) Dynamic rerouting of the carbohydrate flux is key to counteracting oxidative stress. J Biol 6: 10. 18154684 10.1186/jbiol61 PMC2373902

[150] Agarwal AR, Zhao L, Sancheti H, Sundar IK, Rahman I, et al (2012) Short-term cigarette smoke exposure induces reversible changes in energy metabolism and cellular redox status independent of inflammatory responses in mouse lungs. Am J Physiol Lung Cell Mol Physiol 303: L889–898. 23064950 10.1152/ajplung.00219.2012

[151] Magne J, Aminoff A, Perman Sundelin J, Mannila MN, Gustafsson P, et al (2013) The minor allele of the missense polymorphism Ser251Pro in perilipin 2 (PLIN2) disrupts an alpha-helix, affects lipolysis, and is associated with reduced plasma triglyceride concentration in humans. FASEB J 27: 3090–3099. 23603836 10.1096/fj.13-228759

[152] McIntosh AL, Senthivinayagam S, Moon KC, Gupta S, Lwande JS, et al (2012) Direct interaction of Plin2 with lipids on the surface of lipid droplets: a live cell FRET analysis. Am J Physiol Cell Physiol 303: C728–742. 22744009 10.1152/ajpcell.00448.2011 PMC3469596

[153] Bickel PE, Tansey JT, Welte MA (2009) PAT proteins, an ancient family of lipid droplet proteins that regulate cellular lipid stores. Biochim Biophys Acta 1791: 419–440. 19375517 10.1016/j.bbalip.2009.04.002 PMC2782626

[154] Quinones Galvan A, Natali A, Baldi S, Frascerra S, Sanna G, et al (1995) Effect of insulin on uric acid excretion in humans. Am J Physiol 268: E1–5. 7840165 10.1152/ajpendo.1995.268.1.E1

[155] Facchini F, Chen YD, Hollenbeck CB, Reaven GM (1991) Relationship between resistance to insulin-mediated glucose uptake, urinary uric acid clearance, and plasma uric acid concentration. JAMA 266: 3008–3011. 1820474

[156] Bedir A, Topbas M, Tanyeri F, Alvur M, Arik N (2003) Leptin might be a regulator of serum uric acid concentrations in humans. Jpn Heart J 44: 527–536. 12906034 10.1536/jhj.44.527

[157] de Oliveira EP, Burini RC (2012) High plasma uric acid concentration: causes and consequences. Diabetol Metab Syndr 4: 12. 22475652 10.1186/1758-5996-4-12 PMC3359272

[158] Fruehwald-Schultes B, Peters A, Kern W, Beyer J, Pfutzner A (1999) Serum leptin is associated with serum uric acid concentrations in humans. Metabolism 48: 677–680. 10381138 10.1016/s0026-0495(99)90163-4

[159] Osgood K, Krakoff J, Thearle M (2013) Serum Uric Acid Predicts Both Current and Future Components of the Metabolic Syndrome. Metab Syndr Relat Disord 11: 157–162. 23360433 10.1089/met.2012.0151 PMC3661030

[160] Mangge H, Zelzer S, Puerstner P, Schnedl WJ, Reeves G, et al (2013) Uric acid best predicts metabolically unhealthy obesity with increased cardiovascular risk in youth and adults. Obesity (Silver Spring) 21: E71–77. 23401248 10.1002/oby.20061

[161] Lanaspa MA, Sanchez-Lozada LG, Choi YJ, Cicerchi C, Kanbay M, et al (2012) Uric acid induces hepatic steatosis by generation of mitochondrial oxidative stress: potential role in fructose-dependent and -independent fatty liver. J Biol Chem 287: 40732–40744. 23035112 10.1074/jbc.M112.399899 PMC3504786

[162] Rowe PS, Kumagai Y, Gutierrez G, Garrett IR, Blacher R, et al (2004) MEPE has the properties of an osteoblastic phosphatonin and minhibin. Bone 34: 303–319. 14962809 10.1016/j.bone.2003.10.005 PMC3357088

[163] Pfaffl MW (2001) A new mathematical model for relative quantification in real-time RT-PCR. Nucleic Acids Res 29: e45. 11328886 10.1093/nar/29.9.e45 PMC55695

