# Correction: PHEX Mimetic (SPR4-Peptide) Corrects and Improves HYP and Wild Type Mice Energy-Metabolism

**DOI:** 10.1371/journal.pone.0101192

**Published:** 2014-06-18

**Authors:** The PLOS ONE Staff

Figure 3 and Figure 4 are incorrect. The X-axis label should read “Fold Change” and not “% Change”. The authors have provided corrected figures below.

**Figure 3:**
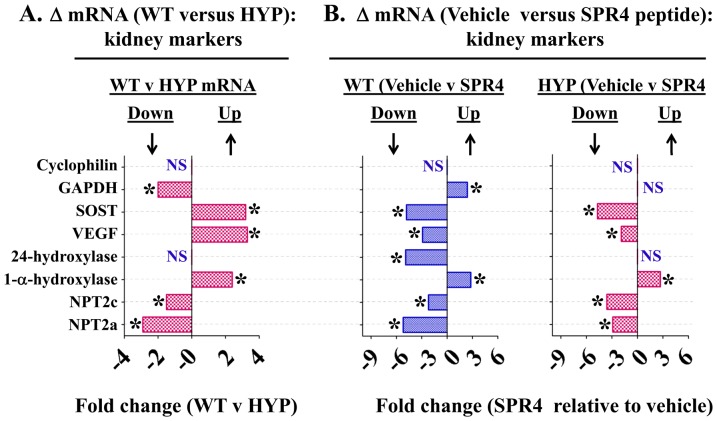
Whole kidney gene expression (mRNA) comparisons as measured by quantitative RT/PCR (qRT-PCR) for wild type (WT) and HYP mice infused with vehicle or SPR4 peptide for 28 days. Column headings represent; WT  =  wild type mice, HYP  =  X-linked hypophosphatemic rickets mice, SPR4  =  infused SPR4-peptide and Vehicle  =  Saline infused. For gene analysis mRNA was prepared from whole kidneys snap frozen in LN2 and homogenized. For qRT-PCR gene analysis fold differences in expression calculated by the Pfaffl method [163] were statistically analyzed for significance using the One Sample t-test and the Wilcoxon Signed rank-test with theoretical means set to 1. Results are significant (*  =  p<0.05) unless indicated by NS (see also **Table 3** for detailed statistics). ND =  Not done, NS  =  Not Significant***Index***: **Cyclophilin**  =  cyclophilin; **GAPDH**  =  Glyceraldehyde 3-phosphate dehydrogenase; **SOST**  =  Sclerostin; **VEGF**  =  Vascular Endothelial Growth factor; **24-Hydroxylase**  =  1,25-hydroxyvitamin D_3_ 24-hydroxylase (CYP24A1); **1-á-Hydroxylase**  =  25-hydroxyvitamin D_3_ 1-alpha-hydroxylase (CYP27B1); **NPT2c**  =  Sodium-dependent phosphate co-transporter (Slc34a3); **NPT2a**  =  Sodium-dependent phosphate co-transporter (Slc34a1); **NS**  =  not significant; *  =  P<0.05. Histogram bars to the left of zero on the axis indicate down regulation and to the right up regulation.

**Figure 4:**
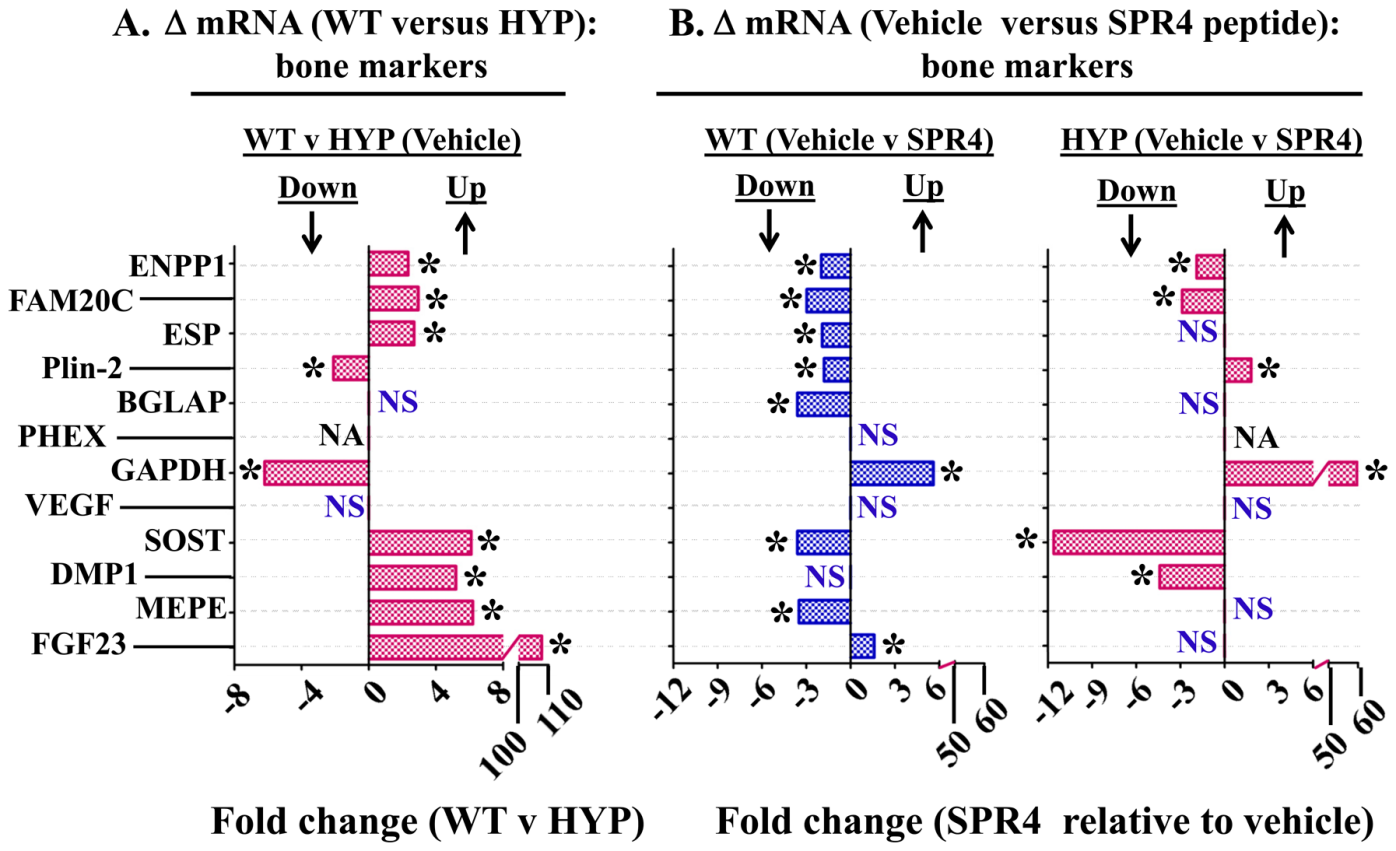
Bone (femur) gene expression (mRNA) comparisons as measured by quantitative RT/PCR (qRT-PCR) for wild type (WT) and HYP mice infused with vehicle or SPR4-peptide for 28 days. Mice were sacrificed on day 28 and femurs collected for RNA purification as described in methods. Column headings represent; WT  =  wild type mice, HYP  =  X-linked hypophosphatemic rickets mice, SPR4  =  infused SPR4-peptide and Vehicle  =  Saline infused. For gene analysis mRNA was prepared from bone marrow stromal cell “*depleted*” femurs as detailed in methods. For qRT-PCR gene analysis fold differences in expression calculated by the Pfaffl method [163] were statistically analyzed for significance using the One Sample t-test and the Wilcoxon Signed rank-test with theoretical means set to 1. Results are significant (* =  p<0.05) unless indicated by NS (see also **Table 4** for detailed statistics). ***Index:*** **FAM20C**  =  Family with sequence similarity 20, member C Kinase also known as DMP4; **ENPP1**  =  Ectonucleotide Pyrophosphatase Phosphodiesterase 1; **ESP**  =  Osteotesticular protein tyrosine (OST-PTP); **Plin-2**  =  Perlipin-2; phosphatase; **Cyclophilin**  =  peptidylprolyl isomerase A (cyclophilin A); **BGLAP**  =  Osteocalcin or Bone Gamma-Carboxyglutamate (gla) protein; **PHEX**  =  Phosphate-regulating gene with Homologies to Endopeptidases on the X chromosome; **GAPDH**  =  Glyceraldehyde 3-phosphate dehydrogenase; **VEGF**  =  Vascular Endothelial Growth factor; **DMP1**  =  Dentin Matrix Protein 1; **SOST**  =  Sclerostin; **MEPE**  =  Matrix Extracellular Phosphoglycoprotein with ASARM -motif; **FGF23**  =  Fibroblast Growth Factor 23; **NS**  =  not significant; **NA**  =  not applicable, PHEX mutated in HYP; *  =  P<0.05. Histogram bars to the left of zero on the axis indicate down regulation and to the right up regulation.
